# Saponin components in *Polygala tenuifolia* as potential candidate drugs for treating dementia

**DOI:** 10.3389/fphar.2024.1431894

**Published:** 2024-07-10

**Authors:** Songzhe Li, Zhitao Hou, Ting Ye, Xiaochen Song, Xinying Hu, Jing Chen

**Affiliations:** ^1^ College of Basic Medicine, Heilongjiang University of Chinese Medicine, Harbin, China; ^2^ The Second Hospital Affiliated Heilongjiang University of Traditional Chinese Medicine, Harbin, China

**Keywords:** triterpenoid saponins, cognitive functions, Alzheimer’s disease, tenuigenin, traditional Chinese medicine, neurodegenerative processes

## Abstract

**Objective:**

This study aims to elucidate the intervention effects of saponin components from *Polygala tenuifolia* Willd (Polygalaceae) on dementia, providing experimental evidence and new insights for the research and application of saponins in the field of dementia.

**Materials and Methods:**

This review is based on a search of the PubMed, NCBI, and Google Scholar databases from their inception to 13 May 2024, using terms such as “*P. tenuifolia*,” “*P. tenuifolia* and saponins,” “toxicity,” “dementia,” “Alzheimer’s disease,” “Parkinson’s disease dementia,” and “vascular dementia.” The article summarizes the saponin components of *P. tenuifolia*, including tenuigenin, tenuifolin, polygalasaponins XXXII, and onjisaponin B, as well as the pathophysiological mechanisms of dementia. Importantly, it highlights the potential mechanisms by which the active components of *P. tenuifolia* prevent and treat diseases and relevant clinical studies.

**Results:**

The saponin components of *P. tenuifolia* can reduce β-amyloid accumulation, exhibit antioxidant effects, regulate neurotransmitters, improve synaptic function, possess anti-inflammatory properties, inhibit neuronal apoptosis, and modulate autophagy. Therefore, *P. tenuifolia* may play a role in the prevention and treatment of dementia.

**Conclusion:**

The saponin components of *P. tenuifolia* have shown certain therapeutic effects on dementia. They can prevent and treat dementia through various mechanisms.

## 1 Introduction

Dementia is a common disabling syndrome characterized primarily by the gradual or progressive loss of memory, executive abilities, and other cognitive functions (Galasko, 2013). As cognitive abilities deteriorate, approximately 90% of individuals with dementia exhibit psychological and behavioral abnormalities, including aggression, psychosis, agitation, and depression (Aarsland, 2020). The decline in cognitive function is typically attributed to neurodegenerative processes, such as the abnormal accumulation of proteins within brain cells and changes in the function of cellular components, which ultimately impair neuronal function and diminish cognitive and memory capacities (Ritchie and Lovestone, 2002). Recent research suggests that vascular aging contributes, at least in part, to the onset of dementia, a finding substantiated in various subtypes of the condition (Andersson and Stone, 2023; Raz et al., 2016). High-risk factors such as hypertension are significant contributors to vascular damage (Lyon et al., 2024). Autopsies indicate that over half of those aged 65 and older exhibit small vessel disease (Hainsworth et al., 2024), underscoring the importance of mitigating risk factors that contribute to cerebrovascular and cumulative brain damage as a strategy for preventing dementia. Dementia is classified into several subtypes, with Alzheimer’s disease (AD) and vascular dementia (VaD) being the most prevalent, accounting for approximately 60% and 20% of cases, respectively. Other forms include Lewy body dementia (about 10%), frontotemporal dementia (about 5%), and Parkinson’s disease dementia (about 2%) (Duong et al., 2017).

According to projections, the global dementia population in 2019 was approximately 55.2 million, with associated societal costs estimated at $1.313 trillion (Wimo et al., 2023). In the United States, the number of Alzheimer’s disease patients aged 65 and older stood at about 6.7 million in 2023, projected to increase to 13.8 million by 2060. The financial burden related to healthcare, long-term care, and end-of-life services is projected to reach $345 billion (Alzheimer’s disease facts and figures, 2023). A further forecast for 2023 estimates the global Alzheimer’s population at 416 million, representing roughly 22% of those aged 50 and over, with 315 million potentially in the preclinical stages of the disease (Gustavsson et al., 2023). Overall, dementia imposes a substantial socio-economic burden, particularly in low- and middle-income countries, where it is estimated that 61% of global dementia costs account for only 26% of total expenditures (Wimo et al., 2023). This disparity likely contributes to a lower quality of life for dementia patients in these regions. The uneven distribution and increasing prevalence of the condition significantly heighten the burden on families and societies (Arruda and Paun, 2017). Given the limited effectiveness of current treatments, there is an urgent need for innovative therapeutic strategies.

*Polygala tenuifolia* (PT) is extensively used in China, Japan, and South Korea as a component of traditional Asian medicine, boasting a long history of application (Jiang et al., 2021; Haraguchi et al., 2022). Ancient Chinese medical texts document PT’s efficacy in treating ailments such as insomnia, neurasthenia, excessive cough with phlegm, and palpitations (Jiang et al., 2021; Chen et al., 2023). In preclinical research, PT and its extracts have demonstrated neuroprotective properties, as well as potential for dementia prevention and antidepressant effects (Shin et al., 2018; Han et al., 2021). Traditional plant-based medicinal combinations containing PT, such as Kaixinsan (Fu et al., 2019), Sagacious Confucius’ Pillow Elixir (Hou et al., 2019), and Buyuan Congnao decoction (Chen et al., 2012a), have also exhibited pharmacological actions against AD. Regarding its chemical composition, PT contains saponins, sugars and glycosides, polygols, ketones, alkaloids, fatty oils, and resins (Tian et al., 2015; Ba et al., 2019; Luo et al., 2024), with several of these constituents confirmed to possess anti-dementia properties. Mechanistically, its actions encompass anti-inflammatory (Zhou et al., 2021), antioxidant (Tang et al., 2022), anti-apoptotic (Choi et al., 2011), anti-amyloid beta protein deposition (Zhao et al., 2015), and neurotransmitter regulation (Lee et al., 2004), providing a broad spectrum of interventions.

Among these, saponin compounds are one of the primary components of *P. tenuifolia*. Saponins are mainly distributed in terrestrial plants, with a diverse array of types and complex structures characterized by their triterpene or steroid aglycone and one or more sugar chains (Güçlü-Ustündağ and Mazza, 2007). Currently, all saponin compounds identified in PT are triterpenoid saponins. Studies have shown that these saponin compounds can penetrate the blood-brain barrier and have a prolonged retention time in the body, providing a material basis for their use in treating brain lesions. Some saponin extracts from PT have been proven to intervene in the progression of dementia through various mechanisms, including anti-inflammatory, antioxidant, anti-apoptotic, and autophagy regulation.

The aim of this review was to examine the pharmacological effects of the saponin active components in PT for the treatment of dementia.

## 2 Saponin compounds in *Polygala tenuifolia*

**FIGURE 1:**
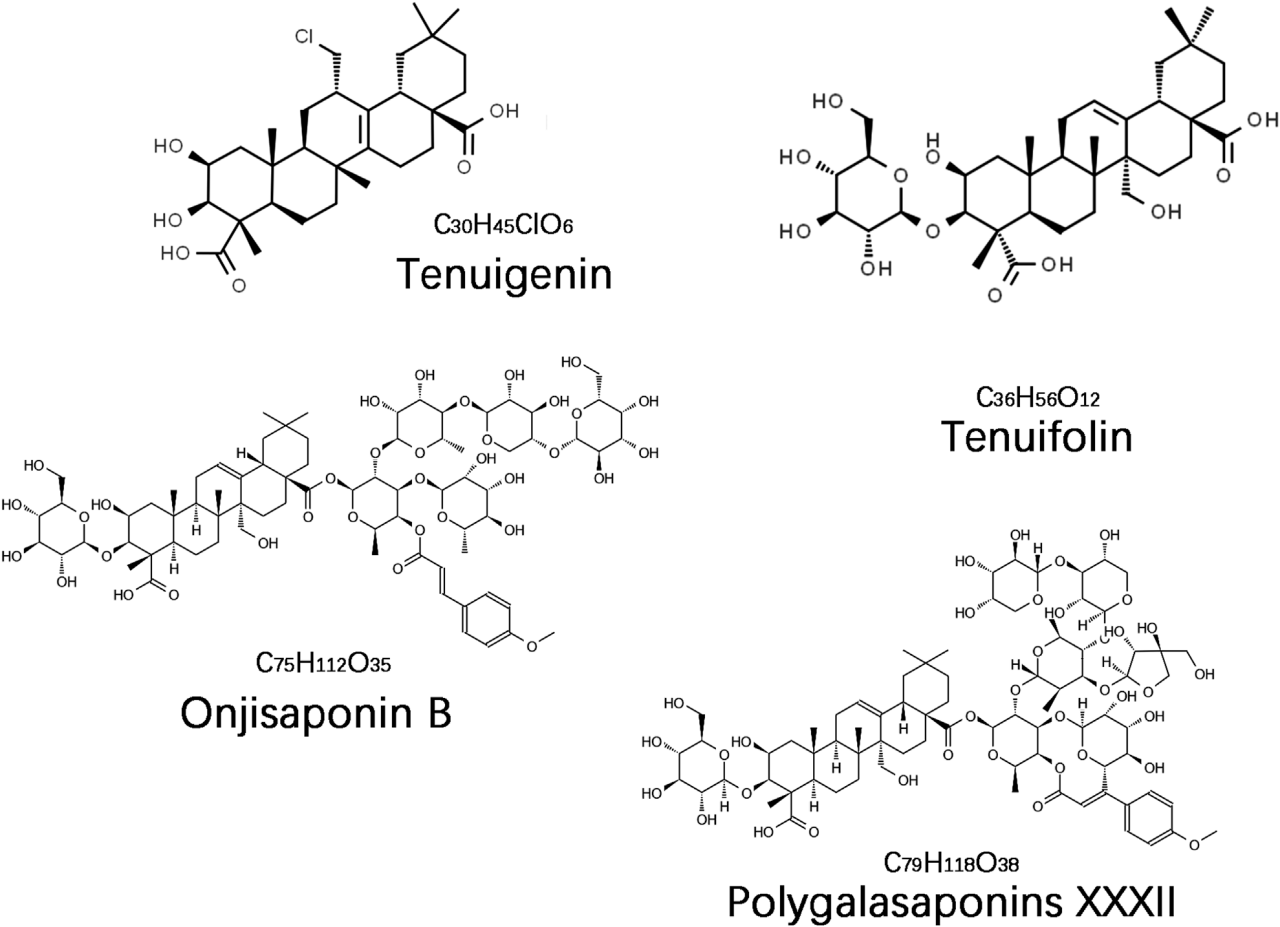
The chemical structure of the main saponin compounds in *Polygala tenuifolia*.

The saponin compounds of *P. tenuifolia* represent some of the most significant active ingredients in PT, all categorized as triterpenoid saponins with a fundamental structure of pentacyclic triterpenes, specifically of the oleanane-type (Liu et al., 2021). To date, numerous PT saponins have been identified, including Sibiricasaponins A-E (Song et al., 2013), onjisaponins (A, B, E, F, etc.) (Zeng et al., 2021), and tenuifoliside A-C (Son et al., 2022). Although extensive, pharmacological research has primarily concentrated on tenuigenin (senegenin), polygalasaponins XXXII, tenuifolin, and onjisaponin B (Figure 1). These saponin compounds have demonstrated extensive neuroprotective effects in the treatment of dementia, employing diverse mechanisms of intervention, and have shown particularly effective outcomes in managing AD and PDD. This review will provide a systematic summary of the action mechanisms of these compounds.

## 3 Plant localization and pharmacokinetics of saponin compounds

The saponin components of PT are predominantly found in the roots, with the concentration in the aboveground parts amounting to only about one-fifth to one-quarter of that in the roots, which is the primary rationale for utilizing the roots for extraction and medicinal purposes (Park et al., 2008; Vinh et al., 2020). Regarding age, there is an inverse relationship between the age of the plant and the concentration of total saponins and sapogenins; older roots contain lower levels of saponins, with annual roots offering the highest quality (Teng et al., 2009). However, the diameter of triennial roots is approximately 2–3 times that of annual roots. Consequently, triennial roots yield a higher biomass, aligning more closely with traditional harvesting practices (Teng et al., 2009).

In terms of pharmacokinetics, saponin compounds exhibit poor membrane permeability and generally demonstrate suboptimal absorption due to physicochemical properties such as a high molecular weight (>500 Da), substantial hydrogen bonding capability (>12), and significant polymer flexibility (>10) (Yu et al., 2012). Tenuigenin, an active ingredient in PT extract, was tested using Institute of Cancer Research (ICR) mice. The half-lives for oral and intravenous administration were recorded at 2.6 ± 0.6 h and 1.6 ± 0.4 h, respectively, with an oral bioavailability of 8.7% (Shen et al., 2022). In contrast, tenuifolin has an oral and intravenous half-life of 1.1 ± 0.2 h and 0.8 ± 0.2 h, respectively, with an oral bioavailability of 4.0% (Shen et al., 2022). When Sprague-Dawley (SD) rats were utilized as research subjects, tenuifolin achieved peak concentrations within 24 min, with terminal elimination half-lives of 4.8 ± 1.6 h and 2.0 ± 0.3 h for oral and intravenous injections, respectively, and an oral bioavailability of 2.0% (Wang et al., 2015). Another study revealed that in SD rats, tenuifolin reached peak concentration 46 min post oral administration, with half-lives of 1.80 ± 0.39 h and 1.41 ± 0.54 h for oral and intravenous injection, respectively, and a bioavailability of 0.83% ± 0.28% (Ma et al., 2014). In terms of tissue distribution, tenuifolin primarily accumulates in the liver and kidneys, with a small amount penetrating the blood-brain barrier and infiltrating brain tissues. The drug’s retention time in various organs does not exceed 12 h (Ma et al., 2014). In summary, PT’s saponin compounds have lower bioavailability but benefit from longer residence times *in vivo* and the ability to penetrate the blood-brain barrier, potentially providing the material basis for treating dementia with PT saponin compounds.

## 4 Pharmacological effects of saponin compounds

**FIGURE 2:**
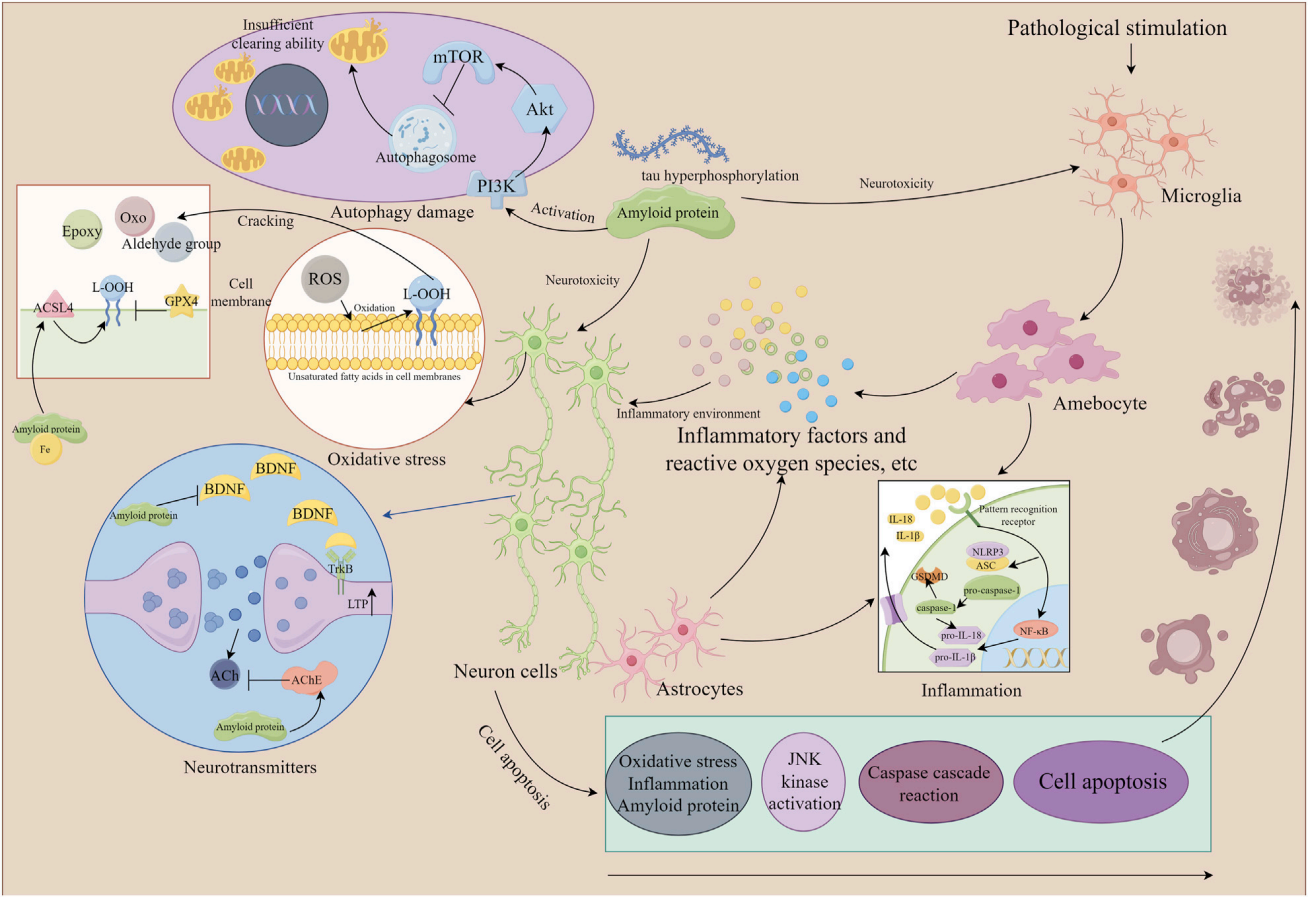
The mechanism of neuronal damage (the part that *Polygala tenuifolia* saponins can intervene in). By Figdraw. Note: ROS, reactive oxygen species; ACSL4, acyl-CoA synthetase long chain family member 4; GPX4, glutathione peroxidase 4; ACh, acetylcholine; JNK, c-Jun N-terminal kinase; NLRP3, recombinant NLR family, pyrin domain containing protein 3; IL, interleukin; NF-κB, nuclear factor-κB; mTOR, mammalian target of rapamycin; BDNF, brain-derived neurotrophic factor; ASC, apoptosis-associated speck-like protein containing a CARD; TrkB, tyrosine kinase receptor B; Akt, protein kinase B; ACSL4, acyl-CoA synthetase long-chain family member 4.

Accumulating evidence indicates that the saponin compounds in PT possess neuroprotective effects, and they are primarily utilized in treating AD within the spectrum of dementia. There is also limited evidence suggesting their potential for interventional in PDD and VaD subtypes. The focus of research on these compounds includes tenuigenin, tenuifolin, polygalasaponins XXXII, and onjisaponin B. Mechanistically, their actions encompass reducing β-amyloid (Aβ) accumulation, providing antioxidant effects, regulating neurotransmitters, enhancing synaptic function, offering anti-inflammatory benefits, preventing neuronal apoptosis, and modulating autophagy (Table 1; Figure 2).

**TABLE 1:** Saponide compounds from *Polygala tenuifolia* in the treatment of dementia.

Active ingredient	Method	Dose	Model	Targets	Actions	References
tenuigenin	*In vitro*	1, 5, 20, 40 μg/mL	PC12 cells	Aβ	Neuroprotection	Jesky and Chen, (2016)
	*In vitro*	15, 30, 60, 120, 240 μM	PC12 cells	ROS, MDA, ACSL4, GPX4, PEBP1	Ferroptosis	Zhang et al. (2022)
	*In vitro*	15, 30, 60, 70, 80 μM	PC12 cells, SD cortical neurons	RhoGDIα, JNK, Bcl-2, Bax	Anti-apoptotic	Li et al. (2015)
	*In vitro*	2, 4 μg/mL	SH-SY5Y cells	Aβ, APP, C99	Targeting Aβ	Jia et al. (2004)
	*In vitro*	1, 2, 4 μg/mL	Primary neural stem cells	Tuj-1, GFAP	Proliferation and differentiation of neural stem cells	Chen et al. (2012b)
	*In vitro*	1, 2, 4 μg/mL	Primary hippocampal neurons	Caspase-3, Bcl-2, Bax, ROS, MAP2	Antioxidant	Chen et al. (2010)
	*In vitro*	10, 20, 40, 60 μM	HT22	LC3-II, LC3-I, PINK1, p62, HSP60, Parkin	Regulating autophagy	Tian et al. (2022)
	*In vivo*	2, 4, 8 mg/kg	SD rats	SOD, GSH-Px, MDA, 4-HNE, p-tau	Inhibition of tau protein phosphorylation	Huang et al. (2018)
	*In vivo*	2 μg/mL	SD rats	—	Synaptic transmission	Wei et al. (2015)
	*In vivo*	4 mg/kg, 2 μg/mL	Kunming mice	SOD, MDA, AChE	Regulation of synaptic plasticity	Huang et al. (2013)
	*In vitro*/*In vivo*	*In vitro*: 2, 4, 8 μM。*In vivo*: 25, 50 mg/kg	BV-2 cells, C57BL/6J mice	ROS, NLRP3, IL-1β, Caspase-1	Anti-inflammatory	Fan et al. (2017)
tenuifolin	*In vitro*	20, 40, 60 μM	Primary neural stem cells	GFAP, NF-M, NG2	Promoting the proliferation of neural stem cells	Wang et al. (2021)
	*In vitro*	0.5, 1, 2 μg/mL	COS-7 cells	Aβ, BACE	Inhibition β- Secretory enzyme	Lv et al. (2009)
	*In vitro*	1, 5, 10 μM	SH-SY5Y cells, BV2 cells	NO, NF-κB, IL-6, IL-1β, TNF-α	Anti-inflammatory	Chen and Jia, (2020)
	*In vitro*	20, 50, 100, 200 μM	SH-SY5Y cells	mTOR, AMPK, ULK1, Beclin-1, LC3-II, LC3-I	Regulating autophagy	Wang et al. (2019b)
	*In vitro*	1, 10, 100, 1000 μM	Primary hippocampal neurons	—	Regulation of synaptic plasticity	Koganezawa et al. (2021)
	*In vivo*	10, 20 mg/kg	ICR mice	SOD, MDA, IL-6, IL-18, IL-1β, IL-10, BDNF, NLRP3, ASC, Caspase-1, HO-1, Nrf2	Antioxidant	Jiang et al. (2023a)
	*In vivo*	0.02, 0.04, 0.08 g/kg	Kunming mice	NE, DA, 5-HT, AChE	Anticholinergic drugs	Zhang et al. (2008)
	*In vivo*	—	Kunming mice	—	Regulation of synaptic plasticity	Kong et al. (2024)
	*In vitro*/*In vivo*	*In vitro*: 1, 5, 10, 20, 40 μM. *In vivo*: 10 mg/kg	HT-22 cells, C57BL/6 mice	SOD, MDA, CAT, GSH, BDNF, TrkB, PSD95, SYN, m-calpain, calpastatin, Akt, ACSL4, GPX4, SLC7A11	Anti-apoptotic, Ferroptosis	Li et al. (2023)
	*In vitro*/*In vivo*	*In vitro*: 10, 20, 40 μg/L. *In vivo*: 3, 9 mg/kg	PC12 cells, C57BL/6J mice	—	Neuroprotection	Liu et al. (2015)
onjisaponin B	*In vitro*	2.5, 5, 10 μM	Primary neural stem cells	GFAP, NF-M, NG2	Proliferation, migration, and differentiation of neural stem cells	Wang et al. (2021)
	*In vitro*	0.1, 1, 10μg/mL	Rat basal forebrain cells	ChAT	Anticholinergic drugs	Yabe et al. (2003)
	*In vitro*	5 μM	PC-12 cells	LC3-II, LC3-I	Regulating autophagy	Wu et al. (2015)
	*In vivo*	10, 20 mg/kg	SD rats	GSH, MDA, SOD, IL-1β, IL-6, TNF-α, NF-κB p65, IκBα	Anti-inflammatory, Antioxidant	Li et al. (2018)
	*In vitro*/*In vivo*	*In vitro*: 10 μM. *In vivo*: 10 mg/kg	HEK293T cells, HEK293 cells, A431 cells, APP/PS1 mice	Aβ, BACE1, γ- Secretory enzyme, APP, C99, C83, sAPPα, sAPPβ, PS1	PS1/BACE1 inhibitors	Li et al. (2016)
Polygala saponins	*In vivo*	50, 100 mg/kg	C57BL/6J mice	SOD, MDA	Antioxidant	Xu et al. (2011)
	*In vivo*	25, 50 mg/kg	SAMP8	NMDAR1, NMDAR2B	Regulation of synaptic plasticity	Xu et al. (2016)
Polygala saponin XXXII	*In vitro*/*In vivo*	*In vitro* *:* 1, 10, 100 μg/mL. *In vivo*: 0.125, 0.5, 2 mg/kg	Primary cortical neurons, PC12 cells, Kunming mice, C57BL/6J mice, Wistar rats	TrkB, p-TrkB	Regulation of synaptic plasticity	Zhou et al. (2016)

Note: Aβ, amyloid β-protein; ROS, reactive oxygen species; MDA, malondialdehyde; ACSL4, acyl-CoA synthetase long chain family member 4; GPX4, glutathione peroxidase 4; PEBP1, phosphatidylethanolamine binding protein 1; APP, amyloid precursor protein; Tuj-1, tubulin beta-III antibody; GFAP, glial fibrillary acidic protein; Bcl-2, B-cell lymphoma-2; Bax, Bcl2-associated X protein; MAP2, microtubule-associated protein 2; LC3, microtubule-associated protein 1A/1B-light chain 3; PINK1, PTEN induced putative kinase 1; p62, sequestosome-1; HSP60, heat shock protein 60; SOD, superoxide dismutase; GSH-Px, glutathione peroxidases; 4-HNE, 4-hydroxynonenal; tau, microtubule-associated protein tau; ACh, acetylcholine; RhoGDIα, Rho guanine nucleotide dissociation inhibitorα; JNK, c-Jun N-terminal kinase; NLRP3, recombinant NLR family, pyrin domain containing protein 3; IL, interleukin; NF-M, neurofilament triplet M; NG2, neural/glial antigen-2; BACE, β-siteAPPcleavingenzyme; NO, nitric oxide; NF-κB, nuclear factor-κB; TNF-α, tumor necrosis factor-α; mTOR, mammalian target of rapamycin; AMPK, adenosine 5‘-monophosphate (AMP)-activated protein kinase; ULK1, unc-51 Like Autophagy Activating Kinase 1; BDNF, brain-derived neurotrophic factor; ASC, apoptosis-associated speck-like protein containing a CARD; HO, heme oxygenase; Nrf2, NF-E2-related factor 2; NE, norepinephrine; DA, dopamine; 5-HT, 5-hydroxytryptamine; CAT, catalase; GSH, glutathione; TrkB, tyrosine kinase receptor B; PSD95, postsynaptic density protein 95; SYN, synaptophysin; Akt, protein kinase B; ACSL4, acyl-CoA synthetase long-chain family member 4; SLC7A11, solute carrier family 7 member 11; IκBα, NF-kappa-B inhibitor alpha; PS1, presenilin-1; NMDAR, N-methyl-daspartate receptor type.

### 4.1 Improving learning and memory

Extracellular amyloid protein deposition and intracellular neurofibrillary tangles are classic pathological hallmarks of Alzheimer’s disease (Bergamino et al., 2022). An imbalance between the generation and degradation of Aβ protein leads to amyloid accumulation (Cai et al., 2023). Aβ protein synthesis is dependent on the β-secretase pathway (von Einem et al., 2010) and is regulated by γ-secretase, with dysregulation of γ-secretase resulting in increased Aβ production (Yang et al., 2021). The primary pathways for Aβ clearance involve glial cell clearance (McAlpine et al., 2021), autophagy (Luo et al., 2020), transport across the blood-brain barrier (Cai et al., 2018), and proteolytic degradation (Zheng et al., 2022). Factors such as an abnormal increase in Aβ protein or aging can impair these clearance mechanisms. Aβ proteins that are not cleared in a timely manner can form oligomers or dispersed amyloid deposits, activating microglia and astrocytes to create a pro-inflammatory environment (Phillips et al., 2014; Franco-Bocanegra et al., 2019), leading to oxidative stress. This stress promotes the excessive phosphorylation of tau proteins, forming neurofibrillary tangles, disrupting microtubule structural stability, causing depolymerization, and ultimately inducing neurotoxicity (Canudas et al., 2005).

Reducing the neuronal toxicity caused by excessive phosphorylation of Aβ and tau proteins is beneficial for ameliorating memory impairment. Studies have demonstrated that tenuigenin and tenuifolin can mitigate Aβ_25-35_-induced neuronal cytotoxicity, thereby enhancing neuronal survival rates (Liu et al., 2015). Furthermore, tenuigenin has been shown to increase the length of neural synapses (Jesky and Chen, 2016). It can also reduce tau protein hyperphosphorylation and oxidative stress levels in the brains of streptozotocin-induced AD rats, thereby intervening in cognitive dysfunction (Huang et al., 2018). Another therapeutic strategy for cognitive impairments involves increasing the number of new neurons and glial cells. Neural stem cells, capable of self-renewal (Andreotti et al., 2019), are located in the subventricular zone of the lateral ventricles and the subgranular zone of the dentate gyrus in humans or mice. These cells possess the potential to differentiate into neurons and astrocytes of the central nervous system. Tenuigenin promotes the proliferation of neural stem cells and induces their differentiation into neurons and astrocytes (Chen et al., 2012b). Both onjisaponin B and tenuifolin enhance the proliferation of neural stem cells, though tenuifolin lacks the capability to facilitate migration and differentiation (Wang et al., 2021), which are crucial for neurogenesis. In contrast, onjisaponin B promotes neural stem cell migration and induces their differentiation into astrocytes (Wang et al., 2021).

### 4.2 Reducing Aβ protein secretion

Excessive secretion of Aβ protein can damage central nervous system cells (Xia et al., 2024). Aβ protein is produced through the enzymatic cleavage of amyloid precursor protein (APP), which is encoded by the *APP* gene located on chromosome 21 (Maurya et al., 2023). APP is a transmembrane protein regulated by two cleavage pathways: the non-Aβ protein generation pathway (Zhao et al., 2022) and the Aβ protein generation pathway (Meng et al., 2023a). Dysregulation of the Aβ protein generation pathway is the primary cause of imbalances in Aβ protein secretion. The β-secretase enzyme is responsible for hydrolyzing and releasing the C-terminal transmembrane peptide segment, C99 (Capone et al., 2021), which is subsequently cleaved by the γ-secretase complex to release Aβ proteins of varying amino acid lengths (Devkota et al., 2024). The most commonly released forms of Aβ protein are Aβ_1-40_ and Aβ_1-42_, with Aβ_1-42_ being more pathogenic and associated with poorer prognosis (Wittrahm et al., 2023).

Inhibiting APP levels and secretase activity can effectively reduce Aβ protein expression and protect central nervous system cells. Tenuigenin reduces APP protein expression by suppressing *APP* mRNA levels in PC12 neural cells, thereby leading to a decrease in the levels of the hydrolysis product C99 (Jia et al., 2004). Further research has revealed that tenuifolin consistently inhibits both Aβ_1-40_ and Aβ_1-42_. However, it does not affect the γ-secretase-mediated cleavage of C99; instead, it reduces Aβ protein expression through the inhibition of β-secretase (Lv et al., 2009). Additionally, presenilin (PS) 1, the catalytic center of γ-secretase, is affected by mutations in the PS1 gene (*PSEN1*), which are a causative factor in early-onset familial Alzheimer’s disease. *PSEN1* mutations increase the expression of β-secretase and γ-secretase, enhancing the sequential cleavage of APP proteins (Li et al., 2022). Onjisaponin B inhibits the interaction between PS1 and β-secretase in APP/PS1 mice, leading to a reduction in Aβ protein secretion, without affecting the activity of β-secretase and γ-secretase (Li et al., 2016).

### 4.3 Antioxidant effect

The imbalance between the production and clearance of reactive oxygen species (ROS) or free radicals leads to oxidative stress, as ROS are natural byproducts of biological oxygen metabolism (Palma et al., 2024). Under normal physiological conditions, ROS stimulate cell growth and rely on antioxidant enzymes to facilitate redox reactions that eliminate excess ROS (Helli et al., 2024). Pathologically, due to the brain’s high oxygen demand and damage to the blood-brain barrier, the transport of ROS and antioxidants both intra- and extracranially becomes challenging (Cornacchia et al., 2022), resulting in an accumulation of ROS within the brain. Furthermore, the neuronal membrane structure, which is rich in unsaturated fatty acids (Yammine et al., 2020), is prone to oxidation. This oxidation, when combined with ROS, forms lipid peroxides and contributes to oxidative damage in the brain. In dementia patients, oxidative stress is closely linked to pathological brain changes. Specifically, Aβ proteins induce neurotoxicity characterized by oxidative stress, oxidizing unsaturated fatty acids on cell membranes to produce lipid peroxides (Koenig and Meyerhoff, 2003), which lead to synaptic loss and ultimately neuron death. Aβ_25-35_ plays a significant catalytic role in this process (Smith and Klimov, 2019). In patients with AD and PDD, there is often an increase in lipid hydroperoxide (L-OOH) and malondialdehyde (MDA) activity, alongside decreased activity of antioxidant enzymes such as superoxide dismutase (SOD), catalase (CAT), and glutathione (GSH) (Chen et al., 2022; Atiq et al., 2023). Moreover, the brain’s rich content of metal ions, such as iron, facilitates oxidative stress and DNA damage through the Haber-Weiss reaction (Suma et al., 2020), and promotes the aggregation of α-synuclein, leading to the formation of Lewy bodies—a key pathological feature of PDD and Lewy body dementia (Wakabayashi et al., 2007). In summary, oxidative stress is an early event leading to cell death, which triggers brain lesions in dementia through programmed cell death processes, such as apoptosis and ferroptosis.

Tenuigenin can reduce the levels of ROS within neuronal cells (Chen et al., 2010). Under pathological conditions, one of the primary sources of ROS is NADPH oxidase, activated when cytokines stimulate cells (Schröder, 2019). Acting as an inhibitor of NADPH oxidase, tenuigenin significantly lowers the accumulation of ROS in PC12 cells (Zhu et al., 2016). Excessive ROS is counteracted by the antioxidant system, with heme oxygenase-1 (HO-1) serving as a crucial metabolic enzyme in responding to oxidative stress. HO-1 is positively regulated by the antioxidant regulatory factor NF-E2 related factor 2 (Nrf2). Upon cellular stimulation, Nrf2 dissociates from Kelch-like ECH-associated protein 1 (Keap1) and translocates from the cytoplasm to the nucleus to activate gene expression. Moreover, HO-1 enhances the expression of glutathione peroxidase (GSH-Px), SOD, and CAT, thus boosting antioxidant capacity (Ryter, 2021). When the antioxidant system fails to effectively suppress ROS, substantial amounts of lipid peroxides are produced, leading to the generation of toxic 4-hydroxynonenal (4-HNE) through non-enzymatic reactions. 4-HNE induces the secretion of Aβ protein, exacerbating cytotoxicity (Takagane et al., 2015). Studies have demonstrated that tenuigenin promotes the nuclear translocation of Nrf2 in neuronal cells, enhances HO-1 expression (Ren et al., 2022), and consequently upregulates the expression of the antioxidant enzyme GSH-Px, while downregulating MDA and 4-HNE levels (Huang et al., 2018). Nitric oxide (NO), another free radical, is positively regulated by inducible nitric oxide synthase (iNOS), which triggers lipid peroxidation by producing peroxynitrite anions (Wan et al., 2021). In microglia, tenuigenin acts as a direct scavenger of NO, although its inhibition of NO is not regulated by iNOS (Lu et al., 2017). Analogously, onjisaponin B reduces oxidative stress in aging rats by downregulating MDA accumulation and upregulating the production of GSH, GSH-Px, and SOD (Li et al., 2018). Tenuifolin elevates the expression of Nrf2 and HO-1 in SH-SY5Y cells, increasing levels of GSH, SOD, and CAT, and reducing MDA and ROS content. Additionally, it suppresses Aβ_42_ oligomer-induced increases in iNOS and NO in microglia (Chen and Jia, 2020). Polygalasaponins enhance SOD activity in the cerebral cortex and hippocampus, lower MDA levels, and inhibit Aβ_25-35_-induced lipid peroxidation and oxidative stress damage (Xu et al., 2011).

### 4.4 Improving synaptic plasticity

The synapse serves as a crucial functional unit of neuronal connectivity, consisting of the presynaptic membrane, synaptic cleft, and postsynaptic membrane. Information is transmitted across these components in sequence, making synaptic plasticity a vital element in facilitating learning and memory functions (Choquet and Triller, 2013). Synaptic plasticity typically manifests as long-term potentiation (LTP) and long-term inhibition, both crucial feedback mechanisms for its regulation. In dementia, LTP is commonly inhibited; thus, stimulating or extending LTP may aid in restoring learning and memory functions (Paoletti et al., 2013). In dementia subtypes such as AD and VaD, synapses display functional deficits, including reductions in synapse numbers, impaired dendritic spine morphology, and diminished neurotransmission capabilities (Piscopo et al., 2022; Che et al., 2023).

Enhancing synaptic morphological changes is beneficial for improving brain information transmission and treating cognitive impairments. Tenuigenin has been shown to improve learning and memory capabilities and offers protective effects on synaptic morphology (Cai et al., 2013). Neurotrophic factors can induce the expression of synaptic-related proteins, such as growth-associated protein-43 (GAP-43), synaptophysin (Syn), and postsynaptic density protein 95, all of which play roles in regulating synaptic plasticity and neurotransmitter release. Tenuigenin enhances the expression of GAP-43 and Syn in PC12 cells induced by Aβ_25-35_, increasing both neurogenesis and the number and length of synapses (Jesky and Chen, 2016). Additionally, tenuigenin affects other proteins involved in memory and synaptic plasticity regulation, reducing the expression of histone deacetylase two and hippocalcin in the rat hippocampus, promoting the phosphorylation of cyclic AMP-responsive element-binding protein and the N-methyl-D-aspartate receptor (NMDAR) 2B subunit, thereby enhancing long-term potentiation (LTP) and synaptic plasticity (Lin et al., 2018). Other compounds, like onjisaponin B, can elevate the expression of neurotrophic factors (Yabe et al., 2003). Tenuifolin directly impacts synaptic status (Koganezawa et al., 2021), increasing the total length and intersection numbers of dendrites in hippocampal CA1 neurons, notably affecting only female mice (Kong et al., 2024). Brain-derived neurotrophic factor (BDNF) is known to activate LTP and increase dendritic spine formation, thus potentially reversing LTP deficits. The tyrosine kinase receptor B (TrkB) serves as a receptor for BDNF, maintaining synaptic protein expression and neuronal survival (Moya-Alvarado et al., 2023). Tenuifolin promotes neuronal cell survival and synaptic protein expression through the BDNF/TrkB signaling pathway (Li et al., 2023). Aβ proteins can inhibit BDNF by reducing the expression of the cyclic AMP response element-binding protein (CREB), while CREB and BDNF-dependent increases promote dendritic growth in neuronal cell bodies. Polyalasaponin XXXII promotes the expression of CREB and BDNF, enhancing the phosphorylation of TrkB and activating downstream cascades, which strengthens synaptic transmission in the dentate gyrus and induces and maintains LTP, enhancing hippocampal synaptic plasticity (Xue et al., 2009; Zhou et al., 2016). NMDAR plays a critical role in regulating synaptic plasticity; under pathological conditions, damage to NMDAR promotes the splicing of APP into Aβ protein, which in turn damages neurons and reduces NMDAR expression (Shankar et al., 2008). Studies have shown that polygalasaponin can reverse the expression of NMDAR subtypes NMDAR1 and NMDAR2B in the hippocampus and cortex of SAMP8 mice, ameliorating cognitive impairments (Xu et al., 2016).

### 4.5 Regulating neurotransmitters

Neurotransmitters are synthesized by presynaptic neurons and released into the synaptic gaps to mediate information transmission between neurons. In dementia patients, neurotransmitter release is typically abnormal (Teleanu et al., 2022; Yang et al., 2023). Over a hundred types of neurotransmitters have been identified, and current research on the effects of saponins from Radix Polygalae primarily focuses on acetylcholine (ACh), 5-hydroxytryptamine (5-HT), norepinephrine (NE), and dopamine (DA). The cholinergic system, closely associated with memory and learning abilities, utilizes choline acetyltransferase (ChAT) as a specific marker for cholinergic neurons. ChAT catalyzes the formation of ACh from choline and acetyl CoA, which then triggers neural impulses in the synaptic gaps, subsequently broken down into choline and acetic acid by acetylcholinesterase (AChE) (Hampel et al., 2018). In dementia, factors such as the loss of cholinergic neurons or deposition of Aβ proteins lead to abnormally elevated levels of AChE (Allard and Hussain Shuler, 2023; Lazarova et al., 2024). Furthermore, 5-HT, DA, and NE, which are closely linked to cognitive functions, when diminished, impair cognitive abilities, manifesting as neuronal damage or metabolic dysfunctions in the brain (Kaneko et al., 2005).

Tenuigenin can enhance learning abilities by inhibiting AChE activity in the hippocampus (Huang et al., 2013) and enhancing field excitatory postsynaptic potentials, which improve synaptic transmission functions (Wei et al., 2015). Onjisaponin B has been shown to strengthen cholinergic neurons and to increase the expression of ChAT (Yabe et al., 2003). Research on tenuifolin indicates that it not only reduces cortical AChE activity but also elevates the expression levels of NE and DA in the hippocampus, although it does not affect the regulation of 5-HT (Zhang et al., 2008). Additionally, NE serves as an anti-inflammatory agent by regulating microglia, astrocytes, and pro-inflammatory factors, thereby reducing brain inflammation (O’Donnell et al., 2012), which underscores tenuifolin’s potential anti-inflammatory effects.

### 4.6 Anti-inflammatory effects

The inflammatory response is intricately linked to the onset and progression of dementia. At early stages of dementia, the brain exhibits high levels of inflammatory responses, primarily involving microglia and astrocytes (Ahmad et al., 2022). Mechanistically, the assembly of inflammasomes is one of the key drivers in the onset and persistence of brain inflammation. Pattern recognition receptors, such as Toll-like receptors, engage with pathogen-related molecular patterns or damage-related molecular patterns (Swanson et al., 2019), leading to the activation of NLR family pyrin domain containing protein 3 (NLRP3) and nuclear factor kappa-B (NF-κB). Upon NLRP3 activation, apoptosis-associated speck-like protein containing a CARD (ASC) and the precursor of pro-caspase-1 are recruited to form an inflammasome complex (Jiang et al., 2023b), subsequently triggering the self-cleavage of pro-caspase-1 to form active caspase-1 (Meng et al., 2023b). Besides activation through pattern recognition receptors, NF-κB is also regulated by pro-inflammatory cytokines such as tumor necrosis factor (TNF)-α and interleukin-1β (IL-1β) (Yu et al., 2020), leading to the transcription of inactive pro-IL-18 and pro-IL-1β (Wang et al., 2023b). Caspase-1 cleaves Gasdermin-D (GSDMD) into GSDMD-N and GSDMD-C, with GSDMD-N binding to the cell membrane to create pores (Meng et al., 2023b). Moreover, caspase-1 cleaves pro-IL-18 and pro-IL-1β into mature IL-18 and IL-1β (Qin and Zhao, 2023), which are released through these membrane pores to promote an inflammatory milieu. Additionally, pro-inflammatory cytokines TNF-α and IL-6 can be directly induced by NF-κB (Yu et al., 2020), further activating NF-κB and perpetuating the release of inflammatory mediators, thus sustaining the inflammatory cycle. The excessive expression of inflammatory factors not only damages neurons and disrupts glial cell function but also causes abnormal glial proliferation, intensifying neurodegenerative changes. Research suggests that certain inflammatory markers such as IL-6 and TNF-α may serve as biomarkers for diagnosing dementia (Darweesh et al., 2018; Custodero et al., 2022).

Microglia are the primary cells responsible for releasing inflammatory mediators in the brain. Under pathological conditions, they serve as a significant source of cytokines and neurotoxic substances. Tenuigenin inhibits lipopolysaccharide-induced microglial activation and the activation of NLRP3 inflammasomes (Fan et al., 2017). The substantia nigra, the principal nucleus responsible for dopamine synthesis, experiences delayed behavioral movements due to dopamine neuron deficiencies and dopamine depletion in the substantia nigra compacta (Kanan et al., 2024). Tenuigenin effectively prevents the degeneration of dopamine neurons and inhibits NLRP3 inflammasome activation in the substantia nigra (Fan et al., 2017). Additionally, Tenuigenin suppresses NF-κB activation in microglia, reducing the cleavage of pro-IL-1β (Li et al., 2017), and decreases the expression of TNF-α, IL-6, and IL-1β (Wang et al., 2017). Moreover, matrix metalloproteinases (MMP) 2 and MMP9, effector factors secreted by microglia, exhibit enhanced expression under the pathological conditions of dementia, promoting neuroinflammation and accelerating neuronal degeneration (Qiu et al., 2023). Studies have shown that tenuigenin reduces the protein expression of MMP2 and MMP9 in primary rat microglia, without affecting the mRNA expression of MMP2 (Lu et al., 2017). Onjisaponin B by inhibiting the NF-κB pathway in the hippocampus, lowers the expression of IL-6, IL-1β, and TNF-α, exerting anti-inflammatory effects (Li et al., 2018). Tenuifolin, by inhibiting the activation of Toll-like receptor 4 and NF-κB in mice with cognitive impairment (Wang et al., 2022), decreases the translation levels of NLRP3, ASC, and caspase-1, reduces inflammasome assembly, and consequently downregulates the expression of IL-6, IL-1β, and IL-18 (Jiang et al., 2023a). In patients with AD, microglia can be activated by Aβ protein, leading to morphological changes and the activation of nitric oxide synthase, which increases NO (Wu et al., 2024) and causes neuronal damage. Cyclooxygenase-2, positively correlated with the density of amyloid plaques, when overexpressed, can worsen cognitive impairments (McLarnon, 2023). Tenuifolin inhibits the Aβ_42_ oligomer-induced inflammatory response in microglia, reducing the release of TNF-α, IL-6, and IL-1β, and suppressing the expression of nitric oxide synthase and cyclooxygenase-2, thus protecting neuronal cells from inflammatory damage (Chen and Jia, 2020).

### 4.7 Anti neuronal apoptosis effect

Apoptosis, a programmed cell death mode, intersects with several dementia pathogenesis hypotheses, including oxidative stress, inflammation, and Aβ-induced cell damage, and represents one of their ultimate outcomes. This process contributes to neuronal loss, which is a pivotal cause of dementia (Liang et al., 2024). Apoptosis is modulated by multiple pathways; notably, saponin components from *P. tenuifolia* primarily inhibit neuronal apoptosis via the c-Jun N-terminal kinase (JNK) pathway, thereby enhancing memory and learning capabilities. Stress stimuli or inflammatory factors can activate JNK kinase, increasing mitochondrial membrane permeability and initiating a caspase cascade reaction (Yuan et al., 2023). The caspase protease family is broadly categorized into initiator caspases (such as caspase-8, 9, and 10) and effector caspases (caspase-3, 6, and 7). Once activated, initiator caspases further activate effector caspases, leading to DNA fragmentation, cytoskeletal and nuclear protein degradation, apoptotic body formation, and subsequent phagocytic uptake (Elmore, 2007). Additionally, JNK pathway activation can regulate the levels of B-cell lymphoma-2 (Bcl-2) family proteins, such as the prototypical anti-apoptotic protein Bcl-2 and the pro-apoptotic protein Bcl2-associated X protein (Bax), which are abnormally expressed in the brains of those with dementia (Rajesh and Kanneganti, 2022).

Recent studies have demonstrated that tenuigenin suppresses caspase-3 activation by downregulating the expression of JNK and phosphorylated JNK in neuronal cells (Chen et al., 2010), thus reversing the expression ratio of Bcl-2 to Bax (Ren et al., 2022). Rho guanine nucleotide dissociation inhibitor α (RhoGDIα), a RhoGDP dissociation inhibitor, participates in the processes of AD and VaD through RhoGTPase activity. Specifically, overexpression of RhoGDIα can reduce Tau hyperphosphorylation and inhibit neuronal apoptosis (Zhang et al., 2023). Tenuigenin elevates the expression of RhoGDIα, which decreases the apoptosis rate in neuronal cells, and this process may be mediated by the JNK pathway (Li et al., 2015). Aβ proteins disrupt intracellular Ca2^+^ distribution, increasing membrane permeability and Ca2^+^ permeability, leading to excessive calpain activation and oxidative stress-induced apoptosis. Tenuifolin inhibits neuronal apoptosis by modulating the calpain system (Li et al., 2023). Furthermore, tenuifolin protects against Aβ_25-35_-induced apoptosis and mitochondrial membrane potential loss in hippocampal neurons, downregulates the expression of caspase-3 and caspase-9, thereby reversing spatial learning and memory deficits in AD mice (Wang et al., 2019a).

### 4.8 Regulating cellular autophagy function

Cellular autophagy is a metabolic regulatory process that recycles and degrades damaged organelles, proteins, and other biomolecules, thereby supporting the growth and development of organisms (Wang et al., 2024). Abnormal regulation of autophagy is commonly observed in dementia patients. In the early stages of AD, there is evident impairment of autophagy, characterized by the earlier aggregation of autophagosomes than Aβ protein deposition, and their failure to be timely degraded by lysosomes (Zhang et al., 2021). As AD progresses, autophagy function significantly weakens, exemplified by the decreased expression of Beclin-1, which is crucial for the formation of autophagic precursors (Bieri et al., 2018). The phosphorylation of intracellular phosphatidylinositol-3-hydroxykinase (PI3K) produces phosphatidylinositol-3,4,5-triphosphate (PIP3), which accumulates on the cell membrane and activates downstream pathways by recruiting and binding proteins such as protein kinase B (Akt) and insulin receptor substrate (IRS) 1 (Zang et al., 2023). A key regulator of autophagy, the mammalian target of rapamycin (mTOR), when activated by Akt, suppresses autophagy by inhibiting the Unc-51 Like Autophagy Activating Kinase 1 (ULK1) complex, preventing the formation of autophagosomes (Kim et al., 2011). In summary, insufficient autophagy flux in the brain may lead to mitochondrial damage and Aβ protein deposition, ultimately impairing cognitive functions.

Aβ_1-42_ can activate the PI3K/Akt signaling pathway in neuronal cells, inhibit autophagy, and subsequently lead to neuronal cell damage. Tenuigenin treatment results in a dose-dependent downregulation of p-PI3K and p-Akt (Ren et al., 2022). Microtubule-associated protein 1A/1B-light chain 3 (LC3)-I is converted to LC3-II through lipid modification. LC3-II is a component of autophagosomes and correlates directly with autophagy flux (Wan et al., 2023). Tenuigenin enhances the expression of LC3-I/LC3-II. Additionally, mitochondrial autophagy is essential for cell survival as it maintains mitochondrial quality by clearing damaged or dysfunctional mitochondria. Under stress conditions, PTEN-induced putative kinase 1 (PINK1) phosphorylates the E3 ubiquitin ligase Parkin, promoting its localization on mitochondrial membranes. This ubiquitination degrades certain mitochondrial proteins and recruits LC3 proteins, which facilitates mitochondrial fusion and the formation of autophagosomes, thereby initiating the autophagy pathway (Eiyama and Okamoto, 2015). Research indicates that tenuigenin upregulates neuronal expression of PINK1 and Parkin, enhances Parkin’s mitochondrial localization, activates autophagy, and increases the rate of autophagic degradation of damaged mitochondria (Tian et al., 2022). Onjisaponin B enhances neuronal autophagy by upregulating the expression of LC3-II (Wu et al., 2015). Tenuifolin improves autophagy levels in neuronal cells induced by Aβ_25-35_ by increasing protein levels of Beclin-1 and LC3-II/I, activating AMP-activated protein kinase (AMPK) and ULK1 expression, and inhibiting mTOR activity to boost autophagy flux. Interestingly, this study also found that Aβ protein itself increases the expression of Beclin-1 and LC3-II/I, possibly reflecting the stress response of neuronal cells in early AD (Wang et al., 2019b).

### 4.9 Maintain iron steady state

Under physiological conditions, central nervous system cells require iron as a cofactor for redox reactions to generate energy. However, oxidative damage can occur when free iron levels increase or antioxidant systems are compromised, ultimately leading to cell ferroptosis (Deng et al., 2023). Elevated iron content has been observed in the brains of patients with several degenerative diseases. Lipid peroxidation acts as a trigger for ferroptosis in cells. Free polyunsaturated fatty acids undergo autooxidation to form L-OOH, and the epoxy, oxo-, or aldehyde groups produced upon L-OOH cleavage are highly toxic oxidative products (Yan et al., 2021). In this context, acyl-CoA synthetase long-chain family member 4 (ACSL4) serves as a critical checkpoint in ferroptosis, facilitating the disease process by catalyzing the formation of L-OOH from arachidonic acid (Tuo et al., 2022). In combating lipid oxidation products, glutathione peroxidase 4 (GPX4) plays a crucial role by detoxifying L-OOH in the membrane, converting toxic L-OOH into non-toxic lipid alcohols, thus significantly mitigating ferroptosis (Reichert et al., 2020).

Some residues of Aβ protein have the ability to bind iron, which upon binding promotes Aβ aggregation and induces neuronal cell death (Wang et al., 2023a). Research has demonstrated that tenuigenin effectively inhibits iron-induced ferroptosis in Aβ_25-35_ stimulated PC12 neurons, mitigating oxidative damage and reversing mitochondrial depolarization. Mechanistically, treatment with tenuigenin reduces the expression of ACSL4 and PEBP1 proteins while elevating GPX4 expression, thereby preventing L-OOH-induced neuronal damage (Zhang et al., 2022). Solute carrier family 7 member 11 (SLC7A11) can be induced under various stress conditions to uptake cysteine, leading to cysteine production and subsequent glutathione synthesis, which supports GPX4 in detoxifying L-OOH (Koppula et al., 2021). The co-administration of d-galactose with Aβ_1-42_ initiates the ferroptosis pathway in HT22 neuronal cells, significantly reducing the expression of L-OOH-associated proteins GPX4 and SLC7A11 and increasing ACSL4 expression. Treatment with tenuifolin reverses these protein expression anomalies and inhibits the ferroptosis process in HT22 cells (Li et al., 2023).

## 5 Toxic effects

PT exhibits inhibitory effects on gastrointestinal peristalsis, potentially leading to intestinal bloating or necrosis (Wen et al., 2015). Further research has demonstrated that onjisaponin B and tenuifolin can act as sub-hypnotic agents, extending the duration of pentobarbital-induced sleep in mice. Additionally, onjisaponin B stimulates gastrointestinal tension in rabbits, inducing irregular contractions of the isolated intestine at an 80 mg/L dosage. Notably, tenuigenin, onjisaponin B, and tenuifolin have all been shown to reduce the expression of prostaglandin E2 (PGE2) in mouse stomachs. PGE2 serves as a gastric mucosal protector, and its reduced expression can compromise this protective effect, leading to gastric damage (Wen et al., 2015). Moreover, polygalasaponins exhibit a synergistic effect with pentobarbital sodium, lengthening sleep duration and decreasing sleep latency (Yao et al., 2010). Regarding acute toxicity, polygalasaponins have a 0% lethal dose of 2.6 g/kg and a median lethal dose of 3.95 g/kg (Yao et al., 2010). In conclusion, the available evidence indicates that PT and its saponin extracts pose certain gastrointestinal toxicities. However, reports on acute, subacute, or long-term toxicity are lacking, making toxicity studies essential for further clinical research. Additionally, since PT saponin components are frequently used in the treatment and research of chronic diseases requiring prolonged medication, defining their safe usage range is crucial.

## 6 Clinical experimental research

According to current reports, there have been no independent clinical studies on PT saponin components. Memantine, an NMDA receptor antagonist, is utilized in treating moderate to severe AD patients (Tang et al., 2023). Research involving 152 AD patients has shown that a 12-week treatment with the combination of tenuigenin (10 mg/d), β-asarone (10 mg/d), and memantine significantly enhances the average Mini-Mental State Examination (MMSE) scores, markedly reduces the Clinical Dementia Rating Scale (CDR) and Activities of Daily Living (ADL) scores, and improves cognitive functions. This combination produces adverse reactions similar to those observed with memantine alone, including mild, transient hallucinations, headaches, nausea, and drowsiness. Additionally, a subgroup analysis identified male patients aged 60–74 with moderate AD as the most beneficial recipients of this treatment regimen (Chang and Teng, 2018). Another clinical study applied the same treatment protocol to 93 AD patients, with increased dosages of tenuigenin (20 mg/d) and β-asarone (20 mg/d) over a 12-week period, resulting in consistent outcomes regarding MMSE, CDR, ADL scores, and side effects (Dong et al., 2018). In conclusion, clinical trials suggest that tenuigenin may have therapeutic effects on AD. However, due to the complexity of the combination therapy, the independent pharmacological impact of tenuigenin requires further validation. Notably, the combination therapy increased symptoms such as drowsiness and nausea compared to using memantine alone (Chang and Teng, 2018), aligning with reports of tenuigenin’s toxicity. Therefore, the possibility that these adverse effects are attributable to tenuigenin cannot be excluded. Future research should intensify preclinical toxicity studies on PT saponin components.

## 7 Discussion

Dementia, as a neurodegenerative disease, exhibits a high incidence rate and a prolonged disease course, imposing substantial psychological and economic burdens on patients and their families (Ayhan et al., 2023). Despite numerous pathogenic hypotheses proposed for its various subtypes, effective medications for treating this condition remain undeveloped, and the pathogenic mechanisms and therapeutic approaches require further exploration. This article provides a comprehensive analysis of the pharmacological effects of the main saponin components of PT in the treatment of dementia, particularly in addressing AD and PDD. The therapeutic potential of PT’s saponins is demonstrated through various mechanisms, including anti-inflammatory, antioxidant, anti-apoptotic, anti-amyloid protein deposition, and neurotransmitter regulation activities. Notably, tenuigenin, onjisaponin B, and tenuifolin exhibit significant neuroprotective effects, contributing to enhanced cognitive function and neuroprotection.

The neuroprotective mechanisms of PT’s saponin components are diverse. Tenuigenin and tenuifolin have been shown to reduce Aβ accumulation, which is central to the pathogenesis of AD. By inhibiting β-secretase activity and modulating γ-secretase, these saponins reduce Aβ production and enhance its clearance through the regulation of microglial activity and autophagy pathways. Additionally, PT’s saponin components mitigate oxidative stress by enhancing the expression of SOD and CAT and by lowering MDA and ROS levels. In terms of neurotransmitter regulation and synaptic plasticity, several saponins from PT enhance cholinergic function by inhibiting AChE activity and increasing ACh levels, thereby improving synaptic plasticity and cognitive function. Moreover, these saponins upregulate the expression of synaptic proteins such as Syn and BDNF, which are crucial for maintaining synaptic integrity and promoting neurogenesis.

However, despite the promising pharmacological effects of PT’s saponin components, their bioavailability remains a significant challenge. Saponin compounds naturally possess characteristics such as high molecular weight, poor membrane permeability, and low oral bioavailability. For instance, tenuigenin and tenuifolin exhibit low oral bioavailability and rapid clearance from the body, necessitating the development of novel delivery systems to enhance their therapeutic efficacy. Clinically, a trial combining tenuigenin with memantine has shown potential benefits in improving cognitive function in AD patients. However, observed side effects, such as gastrointestinal discomfort and sedation, are unfavorable for long-term treatment regimens aimed at slowing disease progression. Therefore, comprehensive toxicological evaluations are required to ensure the safety of long-term use.

Future research should focus on enhancing the bioavailability of PT’s saponin compounds through advanced drug delivery systems such as nanoparticles or liposomes (Kumar and Nair, 2024). Additionally, extensive preclinical toxicity tests and clinical trials are needed to confirm the efficacy and safety of PT’s saponin compounds, whether used as monotherapy or in combination with existing dementia treatments.

## 8 Conclusion

In summary, PT’s saponin compounds show considerable promise in treating dementia through various neuroprotective mechanisms. However, challenges such as low bioavailability and potential side effects must be addressed through further research and clinical validation. The combination of PT’s saponin compounds with existing treatment regimens may offer a novel approach to slowing the progression of neurodegenerative diseases.

## References

[B1] Alzheimer’s disease facts and figures (2023). Alzheimer’s disease facts and figures. Alzheimer’s and Dementia 19, 1598–1695. 10.1002/alz.13016 36918389

[B2] Aarsland D. (2020). Epidemiology and pathophysiology of dementia-related psychosis. J. Clin. Psychiatry 81, 27625. 10.4088/JCP.AD19038BR1C 32936544

[B3] Ahmad M. A. Kareem O. Khushtar M. Akbar M. Haque M. R. Iqubal A. (2022). Neuroinflammation: A potential risk for dementia. International Journal of Molecular Sciences 23, 616. 10.3390/ijms23020616 35054805 PMC8775769

[B4] Allard S. Hussain Shuler M. G. (2023). Cholinergic reinforcement signaling is impaired by amyloidosis prior to its synaptic loss. J. Neurosci. 43, 6988–7005. 10.1523/JNEUROSCI.0967-23.2023 37648452 PMC10586537

[B5] Andersson M. J. Stone J. (2023). Best medicine for dementia: The life-long defense of the brain. J. Alzheimer’s dis. JAD 94, 51–66. 10.3233/JAD-230429 37248910

[B6] Andreotti J. P. Silva W. N. Costa A. C. Picoli C. C. Bitencourt F. C. O. Coimbra-Campos L. M. C. (2019). Neural stem cell niche heterogeneity. Semin. Cell Dev. Biol. 95, 42–53. 10.1016/j.semcdb.2019.01.005 30639325 PMC6710163

[B7] Arruda E. H. Paun O. (2017). Dementia caregiver grief and bereavement: An integrative review. West. J. Nurs. Res. 39, 825–851. 10.1177/0193945916658881 27411975

[B8] Atiq A. Lee H. J. Khan A. Kang M. H. Rehman I. U. Ahmad R. (2023). Vitamin E analog Trolox attenuates MPTP-induced parkinson’s disease in mice, mitigating oxidative stress, neuroinflammation, and motor impairment. International Journal of Molecular Sciences 24, 9942. 10.3390/ijms24129942 37373089 PMC10298414

[B9] Ayhan Y. Yoseph S. A. Miller B. L. (2023). Management of psychiatric symptoms in dementia. Neurol. Clin. 41, 123–139. 10.1016/j.ncl.2022.05.001 36400551

[B10] Ba Y. Wang M. Zhang K. Chen Q. Wang J. Lv H. (2019). Intestinal absorption profile of three polygala oligosaccharide esters in polygalae radix and the effects of other components in polygalae radix on their absorption. Evidence-Based Complementary and Alternative Medicine 2019, 1379531. 10.1155/2019/1379531 31354847 PMC6633864

[B11] Bergamino M. Schiavi S. Daducci A. Walsh R. R. Stokes A. M. (2022). Analysis of brain structural connectivity networks and white matter integrity in patients with mild cognitive impairment. Front. Aging Neurosci. 14, 793991. 10.3389/fnagi.2022.793991 35173605 PMC8842680

[B12] Bieri G. Lucin K. M. O’Brien C. E. Zhang H. Villeda S. A. Wyss-Coray T. (2018). Proteolytic cleavage of beclin 1 exacerbates neurodegeneration. Mol. Neurodegener. 13, 68. 10.1186/s13024-018-0302-4 30594228 PMC6310967

[B13] Cai W. Wu T. Chen N. (2023). The amyloid-beta clearance: From molecular targets to glial and neural cells. Biomolecules 13, 313. 10.3390/biom13020313 36830682 PMC9953441

[B14] Cai Z. Qiao P.-F. Wan C.-Q. Cai M. Zhou N.-K. Li Q. (2018). Role of blood-brain barrier in alzheimer’s disease. Journal of Alzheimer’s disease 63, 1223–1234. 10.3233/JAD-180098 29782323

[B15] Cai Z.-L. Wang C.-Y. Gu X.-Y. Wang N.-J. Wang J.-J. Liu W.-X. (2013). Tenuigenin ameliorates learning and memory impairments induced by ovariectomy. Physiol. Behav. 118, 112–117. 10.1016/j.physbeh.2013.05.025 23688946

[B16] Canudas A. M. Gutierrez-Cuesta J. Rodríguez M. I. Acuña-Castroviejo D. Sureda F. X. Camins A. (2005). Hyperphosphorylation of microtubule-associated protein tau in senescence-accelerated mouse (SAM). Mech. Ageing Dev 126, 1300–1304. 10.1016/j.mad.2005.07.008 16171847

[B17] Capone R. Tiwari A. Hadziselimovic A. Peskova Y. Hutchison J. M. Sanders C. R. (2021). The C99 domain of the amyloid precursor protein resides in the disordered membrane phase. J. Biol. Chem. 296, 100652. 10.1016/j.jbc.2021.100652 33839158 PMC8113881

[B18] Chang W. Teng J. (2018). Combined application of tenuigenin and β-asarone improved the efficacy of memantine in treating moderate-to-severe Alzheimer's disease. Drug Design Development and Therapy 12, 455–462. 10.2147/DDDT.S155567 29551889 PMC5842771

[B19] Che P. Zhang J. Yu M. Tang P. Wang Y. Lin A. (2023). Dl-3-n-butylphthalide promotes synaptic plasticity by activating the akt/ERK signaling pathway and reduces the blood-brain barrier leakage by inhibiting the HIF-1α/MMP signaling pathway in vascular dementia model mice. CNS Neurosci. Ther. 29, 1392–1404. 10.1111/cns.14112 36756709 PMC10068471

[B20] Chen J. Zhang M. Bai H. Shi P. Du M. Zhang S. (2022). Overexpression of C9orf72 exacerbates Aβ25-35-induced oxidative stress and apoptosis in PC12 cells. Acta Neurobiol. Exp. (warsz.) 82, 77–87. 10.55782/ane-2022-007 35451425

[B21] Chen M. Wang J. Ming C. (2012a). Buyuan congnao decoction decreases hippocampal beta-amyloid expression in a rat model of alzheimer’s disease. Neural Regen. Res. 7, 664–668. 10.3969/j.issn.1673-5374.2012.09.004 25745460 PMC4347005

[B22] Chen Q. Jia T. Wu X. Chen X. Wang J. Ba Y. (2023). Polygalae radix oligosaccharide esters may relieve depressive-like behavior in rats with chronic unpredictable mild stress via modulation of gut microbiota. International Journal of Molecular Sciences 24, 13877. 10.3390/ijms241813877 37762181 PMC10530649

[B23] Chen S. Jia J. (2020). Tenuifolin Attenuates Amyloid-β42-Induced Neuroinflammation in Microglia Through the NF-κB Signaling Pathway. J. Alzheimer’s dis. JAD 76, 195–205. 10.3233/JAD-200077 32444542

[B24] Chen Y. Huang X. Chen W. Wang N. Li L. (2012b). Tenuigenin promotes proliferation and differentiation of hippocampal neural stem cells. Neurochem. Res. 37, 771–777. 10.1007/s11064-011-0671-3 22179853

[B25] Chen Y.-J. Huang X.-B. Li Z.-X. Yin L.-L. Chen W.-Q. Li L. (2010). Tenuigenin protects cultured hippocampal neurons against methylglyoxal-induced neurotoxicity. Eur. J. Pharmacol. 645, 1–8. 10.1016/j.ejphar.2010.06.034 20609361

[B26] Choi J. G. Kim H. G. Kim M. C. Yang W. M. Huh Y. Kim S. Y. (2011). Polygalae radix inhibits toxin-induced neuronal death in the parkinson’s disease models. J. Ethnopharmacol. 134, 414–421. 10.1016/j.jep.2010.12.030 21195155

[B27] Choquet D. Triller A. (2013). The dynamic synapse. Neuron 80, 691–703. 10.1016/j.neuron.2013.10.013 24183020

[B28] Cornacchia C. Marinelli L. Di Rienzo A. Dimmito M. P. Serra F. Di Biase G. (2022). Development of l-dopa-containing diketopiperazines as blood-brain barrier shuttle. Eur. J. Med. Chem. 243, 114746. 10.1016/j.ejmech.2022.114746 36099749

[B29] Custodero C. Ciavarella A. Panza F. Gnocchi D. Lenato G. M. Lee J. (2022). Role of inflammatory markers in the diagnosis of vascular contributions to cognitive impairment and dementia: A systematic review and meta-analysis. GeroScience 44, 1373–1392. 10.1007/s11357-022-00556-w 35486344 PMC9213626

[B30] Darweesh S. K. L. Wolters F. J. Ikram M. A. de Wolf F. Bos D. Hofman A. (2018). Inflammatory markers and the risk of dementia and alzheimer’s disease: A meta-analysis. Alzheimer’s and Dementia 14, 1450–1459. 10.1016/j.jalz.2018.02.014 29605221

[B31] Deng L. Mo M.-Q. Zhong J. Li Z. Li G. Liang Y. (2023). Iron overload induces islet β cell ferroptosis by activating ASK1/P-P38/CHOP signaling pathway. Peerj 11, e15206. 10.7717/peerj.15206 37090106 PMC10120586

[B32] Devkota S. Zhou R. Nagarajan V. Maesako M. Do H. Noorani A. (2024). Familial alzheimer mutations stabilize synaptotoxic γ-secretase-substrate complexes. Cell Rep 43, 113761. 10.1016/j.celrep.2024.113761 38349793 PMC10941010

[B33] Dong H. Wu S. Hu N. Xing G. (2018). Efficacy of tenuigenin and β-asarone as augmentations for memantine in the treatment of Alzheimer's disease. Neuroreport 29, 203–207. 10.1097/WNR.0000000000000952 29298173

[B34] Duong S. Patel T. Chang F. (2017). Dementia: what pharmacists need to know. Can. pharm. j. CPJ = Rev. pharm. du Can. RPC 150, 118–129. 10.1177/1715163517690745 PMC5384525 28405256

[B35] Eiyama A. Okamoto K. (2015). PINK1/parkin-mediated mitophagy in mammalian cells. Curr. Opin. Cell Biol. 33, 95–101. 10.1016/j.ceb.2015.01.002 25697963

[B36] Elmore S. (2007). Apoptosis: A review of programmed cell death. Toxicol. Pathol. 35, 495–516. 10.1080/01926230701320337 17562483 PMC2117903

[B37] Fan Z. Liang Z. Yang H. Pan Y. Zheng Y. Wang X. (2017). Tenuigenin protects dopaminergic neurons from inflammation via suppressing NLRP3 inflammasome activation in microglia. J. Neuroinflamm. 14, 256. 10.1186/s12974-017-1036-x PMC5738892 29262843

[B38] Franco-Bocanegra D. K. George B. Lau L. C. Holmes C. Nicoll J. A. R. Boche D. (2019). Microglial motility in Alzheimer's disease and after Aβ42 immunotherapy: a human post-mortem study. Acta Neuropathologica Communications 7, 174. 10.1186/s40478-019-0828-x 31703599 PMC6842157

[B39] Fu H. Xu Z. Zhang X. Zheng G.-Q. (2019). Kaixinsan, a well-known Chinese herbal prescription, for alzheimer’s disease and depression: A preclinical systematic review. Front. Neurosci. 13, 1421. 10.3389/fnins.2019.01421 32009890 PMC6971218

[B40] Galasko D. (2013). The diagnostic evaluation of a patient with dementia. Continuum : Lifelong Learning in Neurology 19, 397–410. 10.1212/01.CON.0000429176.37224.58 23558485 PMC10563933

[B41] Güçlü-Ustündağ O. Mazza G. (2007). Saponins: Properties, applications and processing. Crit. Rev. Food Sci. Nutr. 47, 231–258. 10.1080/10408390600698197 17453922

[B42] Gustavsson A. Norton N. Fast T. Frölich L. Georges J. Holzapfel D. (2023). Global estimates on the number of persons across the alzheimer’s disease continuum. Alzheimer’s and Dementia 19, 658–670. 10.1002/alz.12694 35652476

[B43] Hainsworth A. H. Markus H. S. Schneider J. A. (2024). Cerebral small vessel disease, hypertension, and vascular contributions to cognitive impairment and dementia. Hypertension 81, 75–86. 10.1161/HYPERTENSIONAHA.123.19943 38044814 PMC10734789

[B44] Hampel H. Mesulam M.-M. Cuello A. C. Farlow M. R. Giacobini E. Grossberg G. T. (2018). The cholinergic system in the pathophysiology and treatment of alzheimer’s disease. Brain: J. Neurol. 141, 1917–1933. 10.1093/brain/awy132 PMC6022632 29850777

[B45] Han G. Choi J. Cha S.-Y. Kim B. I. Kho H. K. Jang M.-J. (2021). Effects of radix polygalae on cognitive decline and depression in estradiol depletion mouse model of menopause. Curr. Issues Mol. Biol. 43, 1669–1684. 10.3390/cimb43030118 34698102 PMC8929121

[B46] Haraguchi A. Saito K. Tahara Y. Shibata S. (2022). Polygalae radix shortens the circadian period through activation of the CaMKII pathway. Pharm. Biol. 60, 689–698. 10.1080/13880209.2022.2048863 35298359 PMC8933028

[B47] Helli B. Navabi S. P. Hosseini S. A. Sabahi A. Khorsandi L. Amirrajab N. (2024). The protective effects of syringic acid on bisphenol a-induced neurotoxicity possibly through AMPK/PGC-1α/Fndc5 and CREB/BDNF signaling pathways. Mol. Neurobiol. 10.1007/s12035-024-04048-0 38430353

[B48] Hou Z. Li F. Chen J. Liu Y. He C. Wang M. (2019). Beneficial effects of sagacious confucius’ pillow elixir on cognitive function in senescence-accelerated P8 mice (SAMP8) via the NLRP3/caspase-1 pathway. Evidence-Based Complementary and Alternative Medicine 2019, 3097923. 10.1155/2019/3097923 31781266 PMC6874996

[B49] Huang J. Wang C. Wang X. Wu B. Gu X. Liu W. (2013). Tenuigenin treatment improves behavioral Y-maze learning by enhancing synaptic plasticity in mice. Behav. Brain Res. 246, 111–115. 10.1016/j.bbr.2013.03.001 23499702

[B50] Huang X.-B. Chen Y.-J. Chen W.-Q. Wang N.-Q. Wu X.-L. Liu Y. (2018). Neuroprotective effects of tenuigenin on neurobehavior, oxidative stress, and tau hyperphosphorylation induced by intracerebroventricular streptozotocin in rats. Brain Circulation 4, 24–32. 10.4103/bc.bc_2_17 30276333 PMC6057698

[B51] Jesky R. Chen H. (2016). The neuritogenic and neuroprotective potential of senegenin against Aβ-induced neurotoxicity in PC 12 cells. BMC Complement. Altern. Med. 16, 26. 10.1186/s12906-016-1006-3 26803813 PMC4724108

[B52] Jia H. Jiang Y. Ruan Y. Zhang Y. Ma X. Zhang J. (2004). Tenuigenin treatment decreases secretion of the alzheimer’s disease amyloid beta-protein in cultured cells. Neurosci. Lett. 367, 123–128. 10.1016/j.neulet.2004.05.093 15308312

[B53] Jiang N. Wei S. Zhang Y. He W. Pei H. Huang H. (2021). Protective effects and mechanism of radix polygalae against neurological diseases as well as effective substance. Front. Psychiatry 12, 688703. 10.3389/fpsyt.2021.688703 34975553 PMC8719339

[B54] Jiang N. Zhang Y. Yao C. Liu Y. Chen Y. Chen F. (2023a). Tenuifolin ameliorates the sleep deprivation-induced cognitive deficits. Phytotherapy research: PTR 37, 464–476. 10.1002/ptr.7627 36608695

[B55] Jiang Q. Zhu Z. Mao X. (2023b). Ubiquitination is a major modulator for the activation of inflammasomes and pyroptosis. Biochimica Et Biophysica Acta. Gene Regulatory Mechanisms 1866, 194955. 10.1016/j.bbagrm.2023.194955 37331650

[B56] Kanan M. F. Sheehan P. W. Haines J. N. Gomez P. G. Dhuler A. Nadarajah C. J. (2024). Neuronal deletion of the circadian clock gene Bmal1 induces cell-autonomous dopaminergic neurodegeneration. JCI insight 9, e162771. 10.1172/jci.insight.162771 38032732 PMC10906231

[B57] Kaneko A. Cho S. Hirai K. Okabe T. Iwasaki K. Nanba Y. (2005). Hange-koboku-to, a kampo medicine, modulates cerebral levels of 5-HT (5-hydroxytryptamine), NA (noradrenaline) and DA (dopamine) in mice. Phytother. res. PTR 19, 491–495. 10.1002/ptr.1669 16114091

[B58] Kim J. Kundu M. Viollet B. Guan K.-L. (2011). AMPK and mTOR regulate autophagy through direct phosphorylation of Ulk1. Nat. Cell Biol. 13, 132–141. 10.1038/ncb2152 21258367 PMC3987946

[B59] Koenig M. L. Meyerhoff J. L. (2003). *In vitro* neuroprotection against oxidative stress by pre-treatment with a combination of dihydrolipoic acid and phenyl-butyl nitrones. Neurotox. Res. 5, 265–272. 10.1007/BF03033384 12835118

[B60] Koganezawa N. Sekino Y. Kawakami H. Fuchino H. Kawahara N. Shirao T. (2021). NMDA receptor-dependent and -independent effects of natural compounds and crude drugs on synaptic states as revealed by drebrin imaging analysis. Eur. J. Neurosci. 53, 3548–3560. 10.1111/ejn.15231 33851450 PMC8365428

[B61] Kong H. Han Y.-Y. Yang G.-L. Li K. Yu L. Xie X.-K. (2024). Tenuifolin improves learning and memory by regulating long-term potentiation and dendritic structure of hippocampal CA1 area in healthy female mice but not male mice. Behav. Brain Res. 466, 114974. 10.1016/j.bbr.2024.114974 38554850

[B62] Koppula P. Zhuang L. Gan B. (2021). Cystine transporter SLC7A11/xCT in cancer: Ferroptosis, nutrient dependency, and cancer therapy. Protein and Cell 12, 599–620. 10.1007/s13238-020-00789-5 33000412 PMC8310547

[B63] Kumar V. Nair S. C. (2024). Nano lipid carriers as a promising drug delivery carrier for neurodegenerative disorders - an overview of recent advances. Recent Pat. Biotechnol 18, 2–21. 10.2174/1872208317666230320164219 38205772

[B64] Lazarova M. Tsvetanova E. Georgieva A. Stefanova M. Uzunova D. Denev P. (2024). Extracts of sideritis scardica and clinopodium vulgare alleviate cognitive impairments in scopolamine-induced rat dementia. Int. J. Mol. Sci. 25, 1840. 10.3390/ijms25031840 38339117 PMC10855470

[B65] Lee H. J. Ban J. Y. Koh S. B. Seong N. S. Song K. S. Bae K. W. (2004). Polygalae radix extract protects cultured rat granule cells against damage induced by NMDA. Am. J. Chin. Med. 32, 599–610. 10.1142/S0192415X04002235 15481649

[B66] Li C. Gao F. Qu Y. Zhao P. Wang X. Zhu G. (2023). Tenuifolin in the prevention of alzheimer’s disease-like phenotypes: investigation of the mechanisms from the perspectives of calpain system, ferroptosis, and apoptosis. Phytother. res. PTR, 4621–4638. 10.1002/ptr.7930 37364988

[B67] Li G. Yu J. Zhang L. Wang Y. Wang C. Chen Q. (2018). Onjisaponin B prevents cognitive impairment in a rat model of D-galactose-induced aging. Biomed. Pharmacother. 99, 113–120. 10.1016/j.biopha.2018.01.006 29329033

[B68] Li H. Lin S. Qin T. Li H. Ma Z. Ma S. (2017). Senegenin exerts anti-depression effect in mice induced by chronic un-predictable mild stress via inhibition of NF-κB regulating NLRP3 signal pathway. Int. Immunopharmacol. 53, 24–32. 10.1016/j.intimp.2017.10.001 29031144

[B69] Li N. Qiu Y. Wang H. Zhao J. Qing H. (2022). PS1 affects the pathology of alzheimer’s disease by regulating BACE1 distribution in the ER and BACE1 maturation in the golgi apparatus. International Journal of Molecular Sciences 23, 16151. 10.3390/ijms232416151 36555791 PMC9782474

[B70] Li X. Cui J. Yu Y. Li W. Hou Y. Wang X. (2016). Traditional Chinese Nootropic Medicine Radix Polygalae and Its Active Constituent Onjisaponin B Reduce β-Amyloid Production and Improve Cognitive Impairments. PLoS One 11, e0151147. 10.1371/journal.pone.0151147 26954017 PMC4782990

[B71] Li X. Zhao Y. Liu P. Zhu X. Chen M. Wang H. (2015). Senegenin inhibits hypoxia/reoxygenation-induced neuronal apoptosis by upregulating RhoGDIα. Mol. Neurobiol. 52, 1561–1571. 10.1007/s12035-014-8948-6 25367882

[B72] Liang S. Zhou J. Yu X. Lu S. Liu R. (2024). Neuronal conversion from glia to replenish the lost neurons. Neural Regen. Res. 19, 1446–1453. 10.4103/1673-5374.386400 38051886 PMC10883502

[B73] Lin J. Wang S. Feng Y. Zhao W. Zhao W. Luo F. (2018). Propofol exposure during early gestation impairs learning and memory in rat offspring by inhibiting the acetylation of histone. J. Cell. Mol. Med. 22, 2600–2611. 10.1111/jcmm.13524 29461008 PMC5908131

[B74] Liu L. Feng W.-H. Liu X.-Q. Liang Y.-H. Li C. Wang Z.-M. (2021). research progress on polygalae radix. Zhongguo Zhong Yao Za Zhi 46, 5744–5759. 10.19540/j.cnki.cjcmm.20210518.601 34951162

[B75] Liu Y. Li Z. Hu H. Xu S. Chang Q. Liao Y. (2015). Tenuifolin, a secondary saponin from hydrolysates of polygalasaponins, counteracts the neurotoxicity induced by Aβ25-35 peptides *in vitro* and *in vivo* . Pharmacology, Biochemistry, and Behavior 128, 14–22. 10.1016/j.pbb.2014.11.010 25444865

[B76] Lu L. Li X. Xu P. Zheng Y. Wang X. (2017). Tenuigenin down-regulates the release of nitric oxide, matrix metalloproteinase-9 and cytokines from lipopolysaccharide-stimulated microglia. Neurosci. Lett. 650, 82–88. 10.1016/j.neulet.2017.04.001 28392358

[B77] Luo R. Su L.-Y. Li G. Yang J. Liu Q. Yang L.-X. (2020). Activation of PPARA-mediated autophagy reduces alzheimer disease-like pathology and cognitive decline in a murine model. Autophagy 16, 52–69. 10.1080/15548627.2019.1596488 30898012 PMC6984507

[B78] Luo Y. Hu B. Ji H. Jing Y. Dang X. Zhang H. (2024). Comprehensive evaluation of chemical constituents and antioxidant activity between crude and processed polygalae radix based on UPLC-Q-TOF-MS/MS combined with multivariate statistical analysis. Heliyon 10, e27622. 10.1016/j.heliyon.2024.e27622 38515733 PMC10955230

[B79] Lv J. Jia H. Jiang Y. Ruan Y. Liu Z. Yue W. (2009). Tenuifolin, an extract derived from tenuigenin, inhibits amyloid-beta secretion *in vitro* . Acta Physiol. (Oxf. Engl.) 196, 419–425. 10.1111/j.1748-1716.2009.01961.x 19208093

[B80] Lyon M. Fullerton J. L. Kennedy S. Work L. M. (2024). Hypertension and dementia: Pathophysiology and potential utility of antihypertensives in reducing disease burden. Pharmacol. Ther. 253, 108575. 10.1016/j.pharmthera.2023.108575 38052309

[B81] Ma B. Li X. Li J. Zhang Q. Liu Y. Yang X. (2014). Quantitative analysis of tenuifolin concentrations in rat plasma and tissue using LC⬜MS/MS: Application to pharmacokinetic and tissue distribution study. J. Pharm. Biomed. Anal. 88, 191–200. 10.1016/j.jpba.2013.07.012 24055855

[B82] Maurya R. Bhattacharjee G. Khambhati K. Gohil N. Singh P. Mani I. (2023). Amyloid precursor protein in alzheimer’s disease. Prog. Mol. Biol. Transl. Sci. 196, 261–270. 10.1016/bs.pmbts.2022.09.006 36813361

[B83] McAlpine C. S. Park J. Griciuc A. Kim E. Choi S. H. Iwamoto Y. (2021). Astrocytic interleukin-3 programs microglia and limits alzheimer’s disease. Nature 595, 701–706. 10.1038/s41586-021-03734-6 34262178 PMC8934148

[B84] McLarnon J. G. (2023). Glial-derived neuroinflammation induced with amyloid-beta-peptide plus fibrinogen injection in rat hippocampus. Curr. Alzheimer Res. 20, 515–522. 10.2174/1567205020666230912113501 37702232

[B85] Meng H. W. Kim J.-H. Kim H. Y. Lee A. Y. Cho E. J. (2023a). Paeoniflorin attenuates lipopolysaccharide-induced cognitive dysfunction by inhibition of amyloidogenesis in mice. International Journal of Molecular Sciences 24, 4838. 10.3390/ijms24054838 36902268 PMC10003666

[B86] Meng H. Zhou J. Wang M. Zheng M. Xing Y. Wang Y. (2023b). SARS-CoV-2 papain-like protease negatively regulates the NLRP3 inflammasome pathway and pyroptosis by reducing the oligomerization and ubiquitination of ASC. Microorganisms 11, 2799. 10.3390/microorganisms11112799 38004809 PMC10673202

[B87] Moya-Alvarado G. Tiburcio-Felix R. Ibáñez M. R. Aguirre-Soto A. A. Guerra M. V. Wu C. (2023). BDNF/TrkB signaling endosomes in axons coordinate CREB/mTOR activation and protein synthesis in the cell body to induce dendritic growth in cortical neurons. eLife 12, e77455. 10.7554/eLife.77455 36826992 PMC9977295

[B88] O’Donnell J. Zeppenfeld D. McConnell E. Pena S. Nedergaard M. (2012). Norepinephrine: a neuromodulator that boosts the function of multiple cell types to optimize CNS performance. Neurochem. Res. 37, 2496–2512. 10.1007/s11064-012-0818-x 22717696 PMC3548657

[B89] Palma F. R. Gantner B. N. Sakiyama M. J. Kayzuka C. Shukla S. Lacchini R. (2024). ROS production by mitochondria: Function or dysfunction? Oncogene 43, 295–303. 10.1038/s41388-023-02907-z 38081963

[B90] Paoletti P. Bellone C. Zhou Q. (2013). NMDA receptor subunit diversity: impact on receptor properties, synaptic plasticity and disease. Nat. Rev. Neurosci. 14, 383–400. 10.1038/nrn3504 23686171

[B91] Park H.-J. Lee K. Heo H. Lee M. Kim J. W. Whang W. W. (2008). Effects of polygala tenuifolia root extract on proliferation of neural stem cells in the hippocampal CA1 region. Phytotherapy Research 22, 1324–1329. 10.1002/ptr.2488 18693285

[B92] Phillips E. C. Croft C. L. Kurbatskaya K. O’Neill M. J. Hutton M. L. Hanger D. P. (2014). Astrocytes and neuroinflammation in alzheimer’s disease. Biochem. Soc. Trans. 42, 1321–1325. 10.1042/BST20140155 25233410

[B93] Piscopo P. Crestini A. Carbone E. Rivabene R. Ancidoni A. Lo Giudice M. (2022). A systematic review on drugs for synaptic plasticity in the treatment of dementia. Ageing Res. Rev. 81, 101726. 10.1016/j.arr.2022.101726 36031056

[B94] Qin Y. Zhao W. (2023). Posttranslational modifications of NLRP3 and their regulatory roles in inflammasome activation. Eur. J. Immunol. 53, e2350382. 10.1002/eji.202350382 37382218

[B95] Qiu L. Wang Y. Wang Y. Liu F. Deng S. Xue W. (2023). Ursolic acid ameliorated neuronal damage by restoring microglia-activated MMP/TIMP imbalance *in vitro* . Drug Design Development and Therapy 17, 2481–2493. 10.2147/DDDT.S411408 37637267 PMC10460164

[B96] Rajesh Y. Kanneganti T.-D. (2022). Innate immune cell death in neuroinflammation and alzheimer’s disease. Cells 11, 1885. 10.3390/cells11121885 35741014 PMC9221514

[B97] Raz L. Knoefel J. Bhaskar K. (2016). The neuropathology and cerebrovascular mechanisms of dementia. Journal of Cerebral Blood Flow and Metabolism 36, 172–186. 10.1038/jcbfm.2015.164 26174330 PMC4758551

[B98] Reichert C. O. de Freitas F. A. Sampaio-Silva J. Rokita-Rosa L. Barros P. de L. Levy D. (2020). Ferroptosis mechanisms involved in neurodegenerative diseases. International Journal of Molecular Sciences 21, 8765. 10.3390/ijms21228765 33233496 PMC7699575

[B99] Ren X. Zhang J. Zhao Y. Sun L. (2022). Senegenin inhibits Aβ1-42-induced PC12 cells apoptosis and oxidative stress via activation of the PI3K/akt signaling pathway. Neuropsych. Dis. Treat. 18, 513–524. 10.2147/NDT.S346238 PMC8904946 35280979

[B100] Ritchie K. Lovestone S. (2002). The dementias. Lancet (London, England) 360, 1759–1766. 10.1016/S0140-6736(02)11667-9 12480441

[B101] Ryter S. W. (2021). Heme oxgenase-1, a cardinal modulator of regulated cell death and inflammation. Cells 10, 515. 10.3390/cells10030515 33671004 PMC7997353

[B102] Schröder K. (2019). NADPH oxidase-derived reactive oxygen species: Dosis facit venenum. Exp. Physiol. 104, 447–452. 10.1113/EP087125 30737851 PMC6593456

[B103] Shankar G. M. Li S. Mehta T. H. Garcia-Munoz A. Shepardson N. E. Smith I. (2008). Amyloid-beta protein dimers isolated directly from alzheimer’s brains impair synaptic plasticity and memory. Nat. Med. 14, 837–842. 10.1038/nm1782 18568035 PMC2772133

[B104] Shen X. Dai X. He Y. Wen C. Zhang Q. (2022). Determination of senegenin and tenuifolin in mouse blood by ultra-high performance liquid chromatography-tandem mass spectrometry and their pharmacokinetics. International Journal of Analytical Chemistry 2022, e3401355. 10.1155/2022/3401355 PMC9010192 35432545

[B105] Shin J.-Y. Shin J.-W. Ha S.-K. Kim Y. Swanberg K. M. Lee S. (2018). Radix polygalae extract attenuates PTSD-like symptoms in a mouse model of single prolonged stress and conditioned fear possibly by reversing BAG1. Exp. Neurobiol. 27, 200–209. 10.5607/en.2018.27.3.200 30022871 PMC6050414

[B106] Smith A. K. Klimov D. K. (2019). De novo aggregation of Alzheimer's Aβ25-35 peptides in a lipid bilayer. Sci. Rep. 9, 7161. 10.1038/s41598-019-43685-7 31073226 PMC6509337

[B107] Son S.-R. Yoon Y.-S. Hong J.-P. Kim J.-M. Lee K.-T. Jang D. S. (2022). Chemical constituents of the roots of polygala tenuifolia and their anti-inflammatory effects. Plants (Basel Switz.) 11, 3307. 10.3390/plants11233307 PMC9738712 36501346

[B108] Song Y.-L. Zeng K.-W. Shi T.-X. Jiang Y. Tu P.-F. (2013). Sibiricasaponins a-E, five new triterpenoid saponins from the aerial parts of polygala sibirica L. Fitoterapia 84, 295–301. 10.1016/j.fitote.2012.12.017 23266727

[B109] Suma P. R. Padmanabhan R. A. Telukutla S. R. Ravindran R. Velikkakath A. K. G. Dekiwadia C. D. (2020). Vanadium pentoxide nanoparticle mediated perturbations in cellular redox balance and the paradigm of autophagy to apoptosis. Free Radic. Biol. Med. 161, 198–211. 10.1016/j.freeradbiomed.2020.10.008 33065180

[B110] Swanson K. V. Deng M. Ting J. P.-Y. (2019). The NLRP3 inflammasome: Molecular activation and regulation to therapeutics. Nat. Rev. Immunol. 19, 477–489. 10.1038/s41577-019-0165-0 31036962 PMC7807242

[B111] Takagane K. Nojima J. Mitsuhashi H. Suo S. Yanagihara D. Takaiwa F. (2015). Aβ induces oxidative stress in senescence-accelerated (SAMP8) mice. Biosci. Biotechnol. Biochem. 79, 912–918. 10.1080/09168451.2014.1002449 25612552

[B112] Tang B.-C. Wang Y.-T. Ren J. (2023). Basic information about memantine and its treatment of alzheimer’s disease and other clinical applications. Ibrain 9, 340–348. 10.1002/ibra.12098 37786758 PMC10527776

[B113] Tang X. Zhao Y. Liu Y. Liu Y. Liu Y. Niu F. (2022). 3,6’-disinapoyl sucrose attenuates Aβ1-42 - induced neurotoxicity in caenorhabditis elegans by enhancing antioxidation and regulating autophagy. J. Cell. Mol. Med. 26, 1024–1033. 10.1111/jcmm.17153 35044105 PMC8831957

[B114] Teleanu R. I. Niculescu A.-G. Roza E. Vladâcenco O. Grumezescu A. M. Teleanu D. M. (2022). Neurotransmitters-key factors in neurological and neurodegenerative disorders of the central nervous system. Int. J. Mol. Sci. 23, 5954. 10.3390/ijms23115954 35682631 PMC9180936

[B115] Teng H. Fang M. Cai X. Hu Z. (2009). Localization and dynamic change of saponin in vegetative organs of *polygala tenuifolia* . J. Integr. Plant Biol. 51, 529–536. 10.1111/j.1744-7909.2009.00830.x 19522811

[B116] Tian H. Xu X. Zhang F. Wang Y. Guo S. Qin X. (2015). Analysis of polygala tenuifolia transcriptome and description of secondary metabolite biosynthetic pathways by illumina sequencing. Int. J. Genomics 2015, 782635. 10.1155/2015/782635 26543847 PMC4620389

[B117] Tian Y. Qi Y. Cai H. Xu M. Zhang Y. (2022). Senegenin alleviates Aβ1-42 induced cell damage through triggering mitophagy. J. Ethnopharmacol. 295, 115409. 10.1016/j.jep.2022.115409 35640739

[B118] Tuo Q.-Z. Liu Y. Xiang Z. Yan H.-F. Zou T. Shu Y. (2022). Thrombin induces ACSL4-dependent ferroptosis during cerebral ischemia/reperfusion. Signal Transduction and Targeted Therapy 7, 59. 10.1038/s41392-022-00917-z 35197442 PMC8866433

[B119] Vinh L. B. Heo M. Phong N. V. Ali I. Koh Y. S. Kim Y. H. (2020). Bioactive compounds from polygala tenuifolia and their inhibitory effects on lipopolysaccharide-stimulated pro-inflammatory cytokine production in bone marrow-derived dendritic cells. Plants (Basel, Switzerland) 9, 1240. 10.3390/plants9091240 32962290 PMC7570142

[B120] von Einem B. Schwanzar D. Rehn F. Beyer A.-S. Weber P. Wagner M. (2010). The role of low-density receptor-related protein 1 (LRP1) as a competitive substrate of the amyloid precursor protein (APP) for BACE1. Exp. Neurol. 225, 85–93. 10.1016/j.expneurol.2010.05.017 20685197

[B121] Wakabayashi K. Tanji K. Mori F. Takahashi H. (2007). The lewy body in parkinson’s disease: Molecules implicated in the formation and degradation of alpha-synuclein aggregates. Neuropathology 27, 494–506. 10.1111/j.1440-1789.2007.00803.x 18018486

[B122] Wan W. Qian C. Wang Q. Li J. Zhang H. Wang L. (2023). STING directly recruits WIPI2 for autophagosome formation during STING-induced autophagy. Embo J 42, e112387. 10.15252/embj.2022112387 36872914 PMC10106988

[B123] Wan Y. Song M. Xie X. Chen Z. Gao Z. Wu X. (2021). BMSCs regulate astrocytes through TSG-6 to protect the blood-brain barrier after subarachnoid hemorrhage. Mediators Inflamm 2021, 5522291. 10.1155/2021/5522291 34305453 PMC8263246

[B124] Wang H. Huang H. Jiang N. Zhang Y. Lv J. Liu X. (2022). Tenuifolin ameliorates chronic restraint stress-induced cognitive impairment in C57BL/6J mice. Phytother. Res. 36, 1402–1412. 10.1002/ptr.7402 35129236

[B125] Wang J. Fu J. Zhao Y. Liu Q. Yan X. Su J. (2023a). Iron and targeted iron therapy in alzheimer’s disease. International Journal of Molecular Sciences 24, 16353. 10.3390/ijms242216353 38003544 PMC10671546

[B126] Wang L. Jin G. F. Yu H. H. Lu X. H. Zou Z. H. Liang J. Q. (2019a). Protective effects of tenuifolin isolated from *polygala tenuifolia* willd roots on neuronal apoptosis and learning and memory deficits in mice with alzheimer’s disease. Food Funct 10, 7453–7460. 10.1039/C9FO00994A 31664284

[B127] Wang L. Jin G. Yu H. Li Q. Yang H. (2019b). Protective effect of tenuifolin against alzheimer’s disease. Neurosci. Lett. 705, 195–201. 10.1016/j.neulet.2019.04.045 31039426

[B128] Wang L. Zhang S. Yi S. Ho M. S. (2024). A new regulator of autophagy initiation in glia. Autophagy 20, 207–209. 10.1080/15548627.2023.2251821 37615623 PMC10761159

[B129] Wang P. Tang C.-T. Li J. Huang X. Jin R. Yin F. (2023b). The E3 ubiquitin ligase RNF31 mediates the development of ulcerative colitis by regulating NLRP3 inflammasome activation. Int. Immunopharmacol. 125, 111194. 10.1016/j.intimp.2023.111194 37951199

[B130] Wang Q. Xiao B.-X. Pan R.-L. Liu X.-M. Liao Y.-H. Feng L. (2015). An LC-MS/MS method for simultaneous determination of three polygala saponin hydrolysates in rat plasma and its application to a pharmacokinetic study. J. Ethnopharmacol. 169, 401–406. 10.1016/j.jep.2015.04.033 25922266

[B131] Wang X. Li M. Cao Y. Wang J. Zhang H. Zhou X. (2017). Tenuigenin inhibits LPS-induced inflammatory responses in microglia via activating the Nrf2-mediated HO-1 signaling pathway. Eur. J. Pharmacol. 809, 196–202. 10.1016/j.ejphar.2017.05.004 28478071

[B132] Wang X. Xiao H. Wu Y. Kong L. Chen J. Yang J. (2021). Active constituent of polygala tenuifolia attenuates cognitive deficits by rescuing hippocampal neurogenesis in APP/PS1 transgenic mice. BMC Complementary Medicine and Therapies 21, 267. 10.1186/s12906-021-03437-5 34696749 PMC8543956

[B133] Wei P.-J. Yao L.-H. Dai D. Huang J.-N. Liu W.-X. Xiao P. (2015). Tenuigenin enhances hippocampal schaffer collateral-CA1 synaptic transmission through modulating intracellular calcium. Phytomedicine 22, 807–812. 10.1016/j.phymed.2015.05.008 26220627

[B134] Wen L. Xia N. Tang P. Hong Y. Wang Z. Liu Y. (2015). The gastrointestinal irritation of polygala saponins and its potential mechanism *in vitro* and *in vivo* . Biomed Res. Int. 2015, 918048. 10.1155/2015/918048 25705699 PMC4331466

[B135] Wimo A. Seeher K. Cataldi R. Cyhlarova E. Dielemann J. L. Frisell O. (2023). The worldwide costs of dementia in 2019. Alzheimer’s and Dementia 19, 2865–2873. 10.1002/alz.12901 PMC10842637 36617519

[B136] Wittrahm R. Takalo M. Kuulasmaa T. Mäkinen P. M. Mäkinen P. Končarević S. (2023). Protective alzheimer’s disease-associated APP A673T variant predominantly decreases sAPPβ levels in cerebrospinal fluid and 2D/3D cell culture models. Neurobiol. Dis. 182, 106140. 10.1016/j.nbd.2023.106140 37120095

[B137] Wu A.-G. Wong V. K.-W. Zeng W. Liu L. Law B. Y.-K. (2015). Identification of novel autophagic radix polygalae fraction by cell membrane chromatography and UHPLC-(Q)TOF-MS for degradation of neurodegenerative disease proteins. Sci. Rep. 5, 17199. 10.1038/srep17199 26598009 PMC4657008

[B138] Wu J. Meng Q. Liu D. Fan A. Huang J. Lin W. (2024). Targeted isolation of sorbicilinoids from a deep-sea derived fungus with anti-neuroinflammatory activities. Phytochemistry 219, 113976. 10.1016/j.phytochem.2024.113976 38237844

[B139] Xia X. He X. Zhao T. Yang J. Bi Z. Fu Q. (2024). Inhibiting mtDNA-STING-NLRP3/IL-1β axis-mediated neutrophil infiltration protects neurons in alzheimer’s disease. Cell Prolif 57, e13529. 10.1111/cpr.13529 37528567 PMC10771109

[B140] Xu P. Xu S.-P. Wang K.-Z. Lu C. Zhang H.-X. Pan R. (2016). Cognitive-enhancing effects of hydrolysate of polygalasaponin in SAMP8 mice. J. Zhejiang Univ. Sci. B 17, 503–514. 10.1631/jzus.B1500321 27381727 PMC4940626

[B141] Xu S. P. Yang Y. Y. Xue D. Liu J. X. Liu X. M. Fan T.-P. (2011). Cognitive-enhancing effects of polygalasaponin hydrolysate in aβ(25-35)-induced amnesic mice. Evid.-Based Complement. Altern. Med 2011, 839720. 10.1155/2011/839720 PMC3057668 21423642

[B142] Xue W. Hu J. Yuan Y. Sun J. Li B. Zhang D. (2009). Polygalasaponin XXXII from polygala tenuifolia root improves hippocampal-dependent learning and memory. Acta Pharmacol. Sin. 30, 1211–1219. 10.1038/aps.2009.112 19684611 PMC4007183

[B143] Yabe T. Tuchida H. Kiyohara H. Takeda T. Yamada H. (2003). Induction of NGF synthesis in astrocytes by onjisaponins of polygala tenuifolia, constituents of kampo (japanese herbal) medicine, ninjin-yoei-to. Phytomed. Int. J. Phytother. Phytopharm. 10, 106–114. 10.1078/094471103321659799 12725562

[B144] Yammine A. Nury T. Vejux A. Latruffe N. Vervandier-Fasseur D. Samadi M. (2020). Prevention of 7-ketocholesterol-induced overproduction of reactive oxygen species, mitochondrial dysfunction and cell death with major nutrients (polyphenols, ω3 and ω9 unsaturated fatty acids) of the mediterranean diet on N2a neuronal cells. Molecules (Basel, Switzerland) 25, 2296. 10.3390/molecules25102296 32414101 PMC7287847

[B145] Yan H.-F. Zou T. Tuo Q.-Z. Xu S. Li H. Belaidi A. A. (2021). Ferroptosis: Mechanisms and links with diseases. Signal Transduction and Targeted Therapy 6, 49. 10.1038/s41392-020-00428-9 33536413 PMC7858612

[B146] Yang G. Zhou R. Guo X. Yan C. Lei J. Shi Y. (2021). Structural basis of γ-secretase inhibition and modulation by small molecule drugs. Cell 184, 521–533.e14. 10.1016/j.cell.2020.11.049 33373587

[B147] Yang Z. Zou Y. Wang L. (2023). Neurotransmitters in prevention and treatment of alzheimer’s disease. Int. J. Mol. Sci. 24, 3841. 10.3390/ijms24043841 36835251 PMC9966535

[B148] Yao Y. Jia M. Wu J.-G. Zhang H. Sun L.-N. Chen W.-S. (2010). Anxiolytic and sedative-hypnotic activities of polygalasaponins from *polygala tenuifolia* in mice. Pharm. Biol. 48, 801–807. 10.3109/13880200903280042 20645780

[B149] Yu H. Lin L. Zhang Z. Zhang H. Hu H. (2020). Targeting NF-κB pathway for the therapy of diseases: Mechanism and clinical study. Signal Transduction and Targeted Therapy 5, 209. 10.1038/s41392-020-00312-6 32958760 PMC7506548

[B150] Yu K. Chen F. Li C. (2012). Absorption, disposition, and pharmacokinetics of saponins from Chinese medicinal herbs: What do we know and what do we need to know more? Curr. Drug Metab. 13, 577–598. 10.2174/1389200211209050577 22292787

[B151] Yuan Y. Tan H. Chen H. Zhang J. Shi F. Wang M. (2023). Peroxiredoxin 1 alleviates oxygen-glucose deprivation/reoxygenation injury in N2a cells via suppressing the JNK/caspase-3 pathway. Iran. J. Basic Med. Sci. 26, 1305–1312. 10.22038/IJBMS.2023.71390.15528 37886002 PMC10598809

[B152] Zang L. Fu D. Zhang F. Li N. Ma X. (2023). Tenuigenin activates the IRS1/akt/mTOR signaling by blocking PTPN1 to inhibit autophagy and improve locomotor recovery in spinal cord injury. J. Ethnopharmacol. 317, 116841. 10.1016/j.jep.2023.116841 37355079

[B153] Zeng W. Wu A. G. Zhou X.-G. Khan I. Zhang R. L. Lo H. H. (2021). Saponins isolated from radix polygalae extent lifespan by modulating complement C3 and gut microbiota. Pharmacol. Res. 170, 105697. 10.1016/j.phrs.2021.105697 34062240

[B154] Zhang H. Han T. Zhang L. Yu C.-H. Wan D.-G. Rahman K. (2008). Effects of tenuifolin extracted from radix polygalae on learning and memory: a behavioral and biochemical study on aged and amnesic mice. Phytomed 15, 587–594. 10.1016/j.phymed.2007.12.004 18289838

[B155] Zhang H. Lu F. Liu P. Qiu Z. Li J. Wang X. (2023). A direct interaction between RhoGDIα/tau alleviates hyperphosphorylation of tau in alzheimer’s disease and vascular dementia. Journal of Neuroimmune Pharmacology 18, 58–71. 10.1007/s11481-021-10049-w 35080740

[B156] Zhang H. Zhou W. Li J. Qiu Z. Wang X. Xu H. (2022). Senegenin rescues PC12 cells with oxidative damage through inhibition of ferroptosis. Mol. Neurobiol. 59, 6983–6992. 10.1007/s12035-022-03014-y 36068400

[B157] Zhang Z. Yang X. Song Y.-Q. Tu J. (2021). Autophagy in alzheimer’s disease pathogenesis: Therapeutic potential and future perspectives. Ageing Res. Rev. 72, 101464. 10.1016/j.arr.2021.101464 34551326

[B158] Zhao H. Wang Z.-C. Wang K.-F. Chen X.-Y. (2015). Aβ peptide secretion is reduced by Radix Polygalae-induced autophagy via activation of the AMPK/mTOR pathway. Mol. Med. Rep. 12, 2771–2776. 10.3892/mmr.2015.3781 25976650

[B159] Zhao L. Yue Z. Wang Y. Wang J. Ullah I. Muhammad F. (2022). Autophagy activation by terminalia chebula retz. reduce aβ generation by shifting APP processing toward non-amyloidogenic pathway in APPswe transgenic SH-SY5Y cells. Phytomed. Int. J. Phytother. Phytopharm. 103, 154245. 10.1016/j.phymed.2022.154245 35696798

[B160] Zheng Q. Song B. Li G. Cai F. Wu M. Zhao Y. (2022). USP25 inhibition ameliorates alzheimer’s pathology through the regulation of APP processing and aβ generation. J. Clin. Invest. 132, e152170. 10.1172/JCI152170 35229730 PMC8884900

[B161] Zhou H. Xue W. Chu S.-F. Wang Z.-Z. Li C.-J. Jiang Y.-N. (2016). Polygalasaponin XXXII, a triterpenoid saponin from polygalae radix, attenuates scopolamine-induced cognitive impairments in mice. Acta Pharmacol. Sin. 37, 1045–1053. 10.1038/aps.2016.17 27180981 PMC4973376

[B162] Zhou Y. Yan M. Pan R. Wang Z. Tao X. Li C. (2021). Radix polygalae extract exerts antidepressant effects in behavioral despair mice and chronic restraint stress-induced rats probably by promoting autophagy and inhibiting neuroinflammation. J. Ethnopharmacol. 265, 113317. 10.1016/j.jep.2020.113317 32861821

[B163] Zhu X. Li X. Zhao Y. Ji X. Wang Y. Fu Y. (2016). Effects of senegenin against hypoxia/reoxygenation-induced injury in PC12 cells. Chin. J. Integr. Med. 22, 353–361. 10.1007/s11655-015-2091-8 26759162

